# Large-scale survey, animal models, and computational modeling identify histological neurodegenerative biomarkers for traumatic optic neuropathy

**DOI:** 10.1172/jci.insight.190682

**Published:** 2025-06-17

**Authors:** YiKui Zhang, BoYue Xu, ShiWei Huang, ZhaoHui Shi, Wei Xiong, Ruijun Wang, GuiQin Liu, Linlin Chen, ZhenHua Ge, YongJie Zhang, HongLei Liu, BaoYun Jia, ChunXia Wang, HaiHong Shi, Jun Kang, NingYu An, ShuRui Huang, DeFu Chen, ShengHai Huang, YuTing Luo, MingYue Liu, ZhuoWei Wang, ZhongHao Yu, Jingwei Zheng, Wentao Yan, Gen Li, Hao Chen, XingGuang Deng, ShiHui Wei, YunHai Tu, EnDe Wu, Kang Zhang, WenCan Wu

**Affiliations:** 1 State Key Laboratory of Ophthalmology, Optometry and Vision Science, Eye Hospital, National Clinical Research Center for Ocular Diseases, Eye Hospital, Wenzhou Medical University, Wenzhou, China.; 2 Department of Ophthalmology, the Second Hospital of Jilin University, Changchun, China.; 3 The Third Affiliated Hospital of Sun Yat-Sen University, Guangzhou, China.; 4 The Third Xiangya Hospital of Central South University, Changshao, China.; 5 Li Huili Hospital affiliated with Ningbo University, Ningbo, China.; 6 Shenzhen Eye Hospital, Shenzhen Eye Institute, Jinan University, Shenzhen, China.; 7 The Fourth People’s Hospital of Shenyang, China Medical University, Shenyang, China.; 8 First People’s Hospital of Xuzhou, Xuzhou, China.; 9 Jiaxing Hospital of Traditional Chinese Medicine, Jiaxing, China.; 10 Shananxi Eye Hospital, Xi’an People’s Hospital, Xi’an Fourth Hospital, Affiliated People’s Hospital of Northwest University, Xian, China.; 11 The First Affiliated Hospital of Dali University, Dali, China.; 12 Department of Ophthalmology, The Fourth Affiliated Hospital of China Medical University, Eye Hospital of China Medical University, Key Lens Research Laboratory of Liaoning Province, Shenyang, China.; 13 Eye Institute, Affiliated Hospital of Nantong University, Nantong, China.; 14 Department of Neurosurgery, Beijing Tongren Hospital, Capital Medical University, Tongren, China.; 15 Peoples Hospital of Ningxia Hui Autonomous Region, Yinchuan, China.; 16 Eye Hospital, State Key Laboratory of Eye Health; Institute for Advanced Study on Eye Health and Diseases and Institute for Clinical Data Science, Wenzhou Medical University, Wenzhou, China.; 17 Jiangsu JITRI Sioux Technologies Company, Suzhou, China.; 18 Neuro-Ophthalmology Division, Department of Ophthalmology, Chinese PLA General Hospital, Beijing, China.

**Keywords:** Clinical Research, Ophthalmology, Epidemiology, Neurodegeneration

## Abstract

**BACKGROUND:**

Traumatic optic neuropathy (TON) is a leading cause of blindness following closed traumatic brain injury, with no effective treatments available. Previous interventional clinical trials were complicated by its low prevalence, variability in neurodegenerative severity, and unavailability of reliable biomarkers.

**METHODS:**

We analyzed data from 1,226 patients enrolled in the prospective National Multi-Center Collaborative Clinical Research Program of China (2017–2024) to establish a clinical profile and identify noninvasive biomarkers for neurodegenerative severity. Subgroup analysis of patients with monocular TON revealed potential biomarkers, including visual functional parameters, inner retinal thickness, and time postinjury.

**RESULTS:**

The ganglion cell complex (GCC) thickness showed a strong correlation with retinal ganglion cell somata (*R*² = 0.87, *P* < 0.0001) and axon density (*R*² = 0.89, *P* < 0.0001) in a clinically relevant large animal model. Computational analysis demonstrated that using GCC thickness as a biomarker could substantially enhance the statistical power of clinical trials (by up to 4.5-fold), as verified by real-world data.

**CONCLUSION:**

This study presents the largest epidemiological analysis of TON to date and establishes GCC thickness as a crucial biomarker for stratifying disease severity and improving the efficiency of clinical trials.

**TRIAL REGISTRATION:**

Chinese Clinical Trial Registry (ChiCTR-OOC-17013437).

**FUNDING:**

National Key R&D Program of China (Grant No. 2022YFA1105500), Key Science and Technology Program of Wenzhou (Grant No. ZY2022021), National Natural Science Foundation of China (Grant No. 82471080).

## Abstract 2

This large prospective observational study of traumatic optic neuropathy identifies inner retinal thickness as a key biomarker for disease severity assessment and clinical trials design.

## Introduction

Traumatic optic neuropathy (TON) is a major form of visual pathway injury that occurs after blunt traumatic brain injury (TBI) (1) and leads to devastating vision loss. It is observed in up to 40%–72% of patients with TBI who experience loss of consciousness (2–4). In a study examining 84 autopsies of acute closed head injury cases, interstitial hemorrhage and axonal lesions in the optic nerve (ON) were found in 36% and 44% of patients, respectively (5). A majority of patients with TON (65%–84%) present with visual acuity (VA) of their injured eyes worse than 20/200, the threshold for legal blindness (6–10). Although approximately 50% of cases might show spontaneous improvement in visual function following presentation (7, 9, 11, 12), most patients with TON (69%–78%) experience permanent legal blindness in their injured eyes (11, 13, 14).

The importance of successful clinical trials for TON has been further underscored by the recent launch of the Transplantation of Human Eye Allografts (THEA) program by the Advanced Research Projects Agency for Health in 2024. The ambitious goal of THEA to restore vision through whole-eye transplantation critically depends on effective ON repair and eye-brain reconnection processes, for which TON serves as the predominantly used model in basic research. Although there have been numerous demonstrations of the preclinical efficacy of therapeutics targeting ON protection and regeneration in TON animal models (15, 16), TON clinical trials performed to date have failed to demonstrate consistent therapeutic efficacy for any intervention (4, 17, 18). Factors hindering the success of these trials include low annual incidence rates (approximately 1 case per million in the general population), complex clinical presentations, and high variability in neurodegenerative severity at presentation (6, 10).

Identifying reliable neurodegenerative biomarkers is crucial for overcoming these challenges. Effective TON biomarkers can enable stratification by disease severity, improve clinical trial efficiency, reduce required clinical trial sizes, and reduce study duration. While the densities of retinal ganglion cell (RGC) somata and axons along the ON are considered benchmarks for grading neurodegenerative severity in preclinical animal studies, these histological measurements are inaccessible in the clinical context. Previous trials have relied primarily on visual function outcomes, such as VA (2–4, 7, 9, 11, 12, 17) (Supplemental Table 1; supplemental material available online with this article; https://doi.org/10.1172/jci.insight.190682DS1). However, visual function alone is insufficient for evaluating the severity of neurodegeneration in TON, as similar axonal dysfunction can occur at different stages of disease progression (Supplemental Figure 1).

To address these critical issues, we conducted the largest survey of TON to date, analyzing data from 1,226 patients enrolled in China’s National Multi-Center Collaborative Clinical Research Program (2017–2024), and characterized the demographics and clinical profiles of TON. We identify noninvasive biomarkers, particularly inner retinal thickness, which correlate strongly with RGC somata and axon densities in large animal models. Through computational modeling and real-world data analysis, we investigate how integrating these biomarkers may affect clinical trial design for TON. This study aims to improve the design and effectiveness of future clinical trials in TON, with potential impact on both the THEA program and clinical trial designs for other optic neuropathies.

## Results

### Epidemiology, clinical features, and estimated annual incidence of TON.

**Figure 1:**
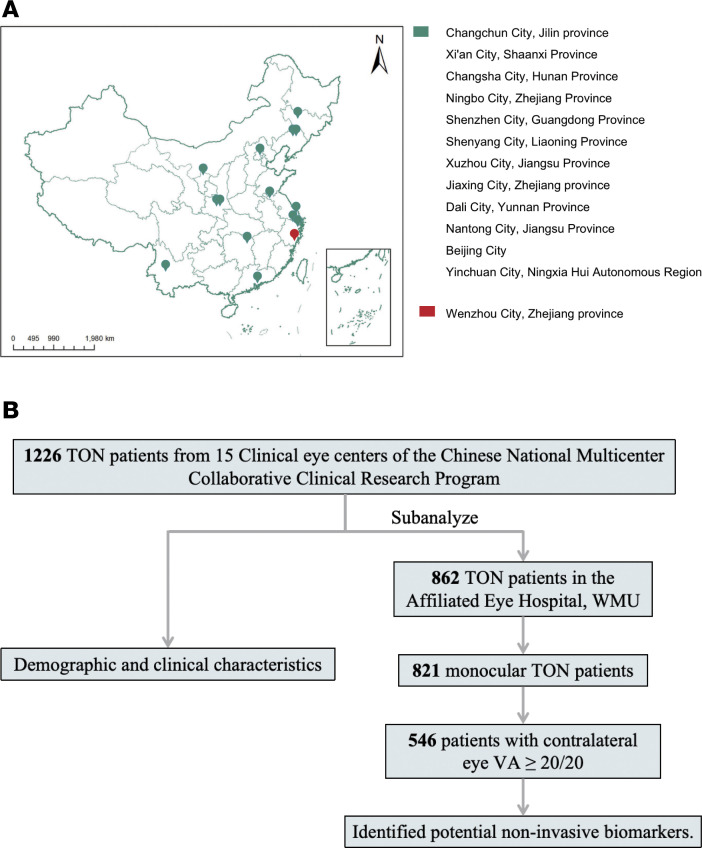
Distribution of eye clinics and flowchart of patient enrollment in the National Multi-Center Collaborative program for TON in mainland China. (**A**) Geographical distribution of participating clinical eye centers across mainland China. (**B**) Flowchart detailing patient enrollment and data analysis. Changchun City, Jilin Province (The Second Norman Bethune Hospital of Jilin University, *n* = 100). Xi’an City, Shaanxi Province (The First Affiliated Hospital of AFMU, *n* = 31; Xian People’s Hospital, *n* = 18). Changsha City, Hunan Province (The Third Xiangya Hospital of Central South University, *n* = 30). Ningbo City, Zhejiang Province (Ningbo Medical Center Lihuili Hospital, *n* = 30). Shenzhen City, Guangdong Province (Shenzhen Eye Hospital, *n* = 28). Shenyang City, Liaoning Province (The Fourth People’s Hospital of Shenyang, CMU, *n* = 25; The Fourth Affiliated Hospital of China Medical University, *n* = 17). Xuzhou City, Jiangsu Province (The First People’s Hospital of Xuzhou, *n* = 20). Jiaxing City, Zhejiang Province (Jiaxing Hospital of Traditional Chinese Medicine, *n* = 18). Dali City, Yunnan Province (The First Affiliated Hospital of Dali University, *n* = 17). Nantong City, Jiangsu Province (Affiliated Hospital of Nantong University, *n* = 14). Beijing City (Beijing Tongren Hospital, CMU, *n* = 9). Yinchuan City, Ningxia Hui Autonomous Region (People’s Hospital of Ningxia Hui Autonomous Region, *n* = 7). WMU, Wenzhou Medical University; VA, visual acuity.

A total of 1,226 patients from 15 clinical eye centers in mainland China were included in the overall analysis (Figure 1A). The processes of patient enrollment and clinical data collection are depicted (Figure 1B). Among the enrolled patients, 83.6% were male. The ages of the patients ranged from 0 to 83 years, with 67.7% of them aged between 18 and 60 years, referred to as the working-age population. The median age and IQR of the patients both were 33 years. The median time from injury to ophthalmic examination was 13 days (IQR: 28 days) across all 15 clinical eye centers (Table 1). At Wenzhou Eye Hospital, WMU, where 92.6% of patients with TON came from outside Wenzhou, the time interval was 18 days (IQR: 43 days), compared with 7 days (IQR: 15 days) at other local centers.

**Figure 2:**
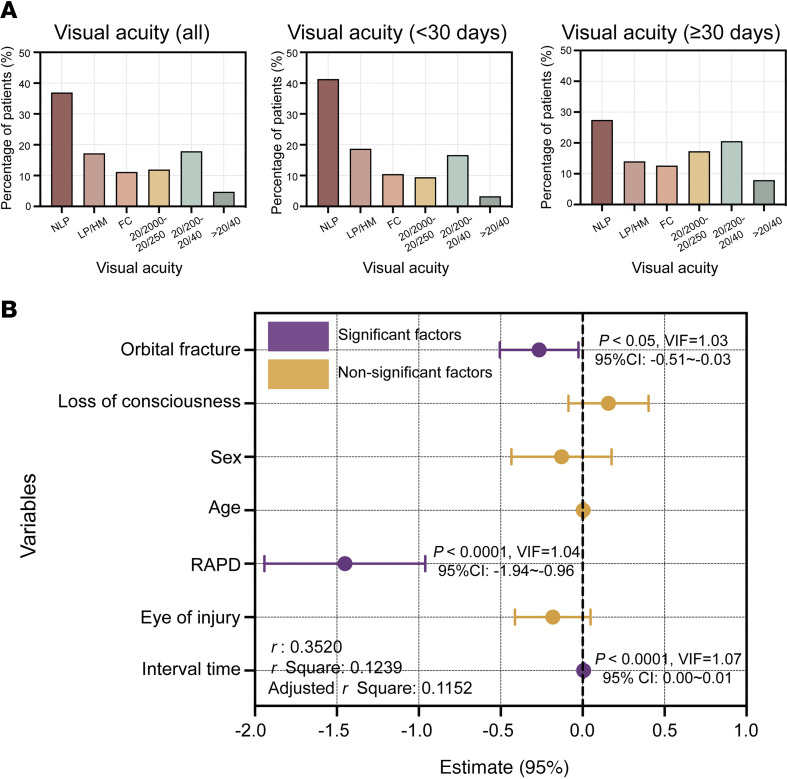
Distribution of VA and associated factors in the injured eye of patients with TON. (**A**) Distribution of best-corrected visual acuity in patients with TON. Left panel: All patients. Middle panel: Patients hospitalized within 30 days after injury (*n* = 791). Right panel: Patients hospitalized ≥30 days after injury (*n* = 364). (**B**) Factors associated with best-corrected visual acuity. The *x* axis (estimate [95% CI]) indicates the size and direction of each variable’s effect on the logMAR VA (*n* = 707). Statistical significance was considered at *P* < 0.05 by multiple linear regression for **B**. VIF, variance inflation factor; RAPD, relative afferent pupillary defect; NLP, no light perception; MAR, minimum angle of resolution; LP, light perception; HM, hand motion; GCC, ganglion cell complex FC, finger count.

Most patients had monocular TON (96.3%). The most common cause of TON was falling from a height (45.7%), followed by motor vehicular accidents (33.4%) and blunt assault (11.2%). Orbital fractures were found in 60.4% of patients. Intracranial injury (such as intracranial hemorrhage and skull fracture) and transient coma following trauma were reported in 31.8% and 28.6% of patients, respectively (Table 1). Most patients experienced severe visual loss in their injured eyes (Figure 2A). Approximately 77.3% of all the patients presented with a best-corrected VA (VAcc) in the injured eye worse than 20/200, which is the threshold for international legal blindness. Among patients who presented to eye clinics 1 month or more after injury, 71.4% of them had a VAcc worse than 20/200. Multiple linear regression analysis revealed that VA was significantly associated with orbital fracture (β = –0.27, 95% CI, –0.51~–0.03, *P* < 0.0001) and relative afferent pupillary defect (RAPD) positivity (β = –1.45, 95% CI, –1.94~–0.96, *P* < 0.001) and was not significantly associated with age, sex, the eye of injury, and history of coma. Although the results indicated a significant correlation between VA and the time interval between injury and ophthalmic examination (β = 0.01,95% CI, 0.00 –0.01, *P* < 0.05), the impact of time on the extent of vision loss following TON was minimal (Figure 2B).

Based on the number of TON cases seen at Wenzhou Eye Hospital, the estimated minimal annual incidence of TON in Wenzhou City (with a population of 9 million) was approximately 1 case per year per 1 million population between 2018 and 2024, similar to the rate reported in the United Kingdom a decade ago (10). During the COVID-19 pandemic (2019 and 2020), the TON incidence in Wenzhou City sharply decreased, likely owing to partial lockdown measures (Supplemental Figure 2). It is important to note that the incidence may have been underestimated for 2 reasons: the study only included patients who were conscious and able to communicate, and not all local patients with TON in Wenzhou were referred to this specialized eye hospital, particularly those with severe head trauma.

### Potential biomarkers for assessing the severity of neurodegeneration in monocular TON.

To detect potential biomarkers that may be associated with neurodegenerative severity, we conducted a subgroup analysis in 546 patients. The inclusion criteria for this subgroup included monocular injury and a VAcc of 20/20 or better in the contralateral, uninjured eyes, which were confirmed to have normal visual field (VF), fundus imaging, and optical coherence tomography (OCT) results, with no eye-specific comorbidities such as branch retinal vein occlusion or diabetic retinopathy, ensuring their reliability as controls (Supplemental Figure 3 and Supplemental Table 2). The visual functional parameters — such as VA, pupillary light reflex (PLR), mean deviation (MD), and visual field index (VFI) from automatic static perimetry and P100 amplitude and latency from pattern visual evoked potential (P-VEP) — and structural parameters — retinal nerve fiber layer (RNFL) or ganglion cell complex (GCC) layer thickness from OCT — were compared between the injured and contralateral uninjured eyes.

**Figure 3:**
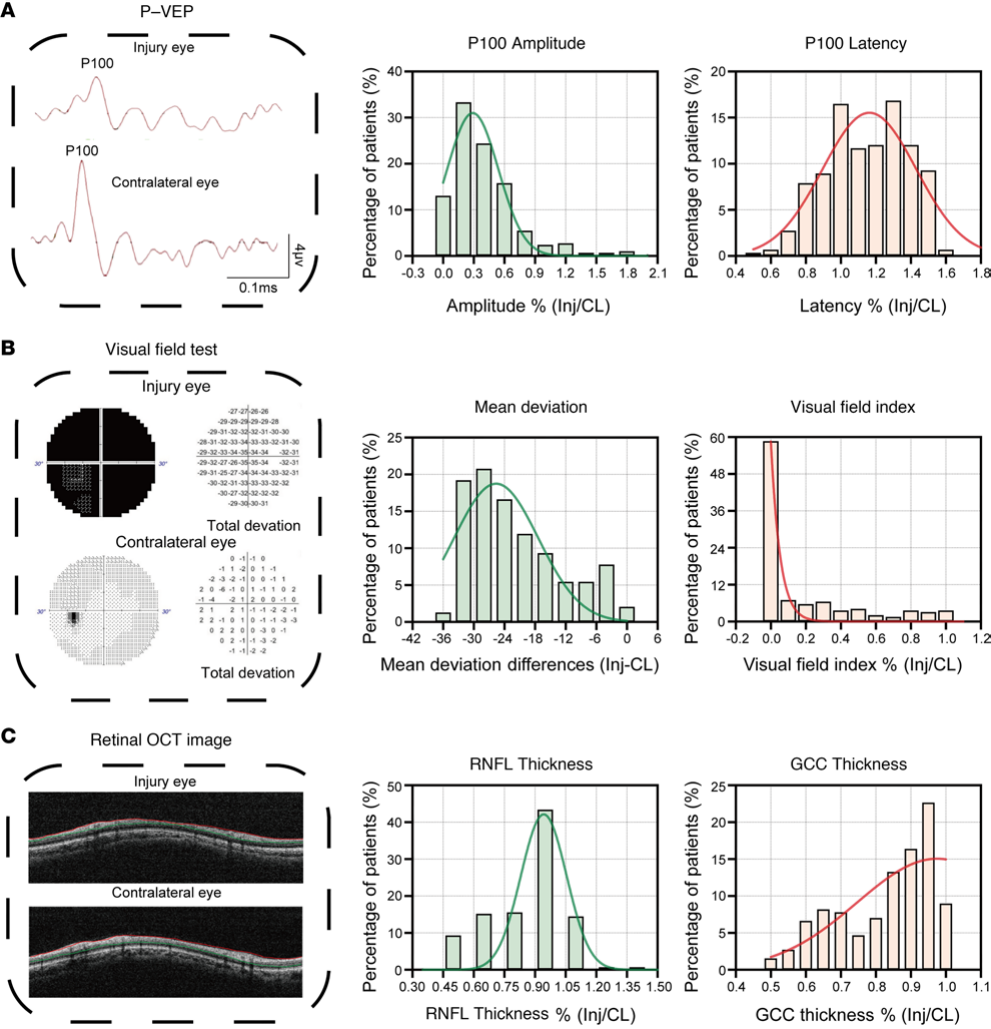
Distribution of functional and structural deficits in patients with monocular TON. (**A**) Representative P-VEP waveforms comparing the injured and contralateral eyes in patients with monocular TON (left panel). Histograms showing distributions of P100 amplitude ratios (middle panel) and latency ratios (right panel) between the injured eyes and the contralateral eyes in patients with monocular TON (*n* = 291). (**B**) Representative VF test results comparing the injured and contralateral eyes in patients with monocular TON (left panel). Histograms showing distributions of MD difference (middle panel) and VFI ratio (right panel) between the injured and contralateral eyes in patients with monocular TON (*n* = 385). (**C**) Representative retinal OCT images comparing the injured and contralateral eyes in patients with monocular TON (left panel). Histograms showing distributions of RNFL (middle panel, *n* = 546) and GCC thickness ratios (right panel, *n* = 264) between the injured and contralateral eyes in patients with monocular TON. Smooth curve derived from Gaussian nonlinear regression fit for all histogram panels. P-VEP, pattern visual evoked potential; OCT, optical coherence tomography; GCC, ganglion cell complex; RNFL, retinal nerve fiber layer; MD, mean deviation; VFI, visual field index; Inj, injury; CL, contralateral.

Patients with monocular TON exhibit varying degrees of functional and structural deficits in the ON following injury. The mean VA of the injured eyes was significantly worse than that of the contralateral eyes (mean ± SD: log minimum angle of resolution [logMAR], –2.54 ± 1.72 vs. 0.00 ± 0.02; *P* < 0.0001). Consistently, P100 amplitude was significantly lower in the injured eye (median [IQR], 3.20 [1.70 to 6.50] μv vs. 6.10 [2.60 to 10.10] μv; *P* < 0.0001), and P100 latency was significantly longer in the injured eye (median [IQR], 120.00 [102.00 to 143.00] ms vs. 104.00 [100.00 to 108.00] ms; *P* < 0.0001). The MD was lower in the injured eye than the control eye (median [IQR], −30.17 [−33.13 to –21.18] dB vs. –3.15 [−5.46 to –1.67] dB; *P* < 0.0001). The VFI was also much lower in the injured eye (median [IQR], 0.00 [0.00 to 28.00] vs. 97.00 [94.00 to 99.00] dB; *P* < 0.0001). The RNFL was significantly thinner in the injured eye (median [IQR], 96.00 [74.00 to 107.00] μm vs. 106.00 [100.00 to 112.00] μm; *P* < 0.0001). Similarly, the GCC thickness was significantly thinner in the injured eye compared with that in the contralateral eye, as indicated by the relative GCC thickness percentage between the injured and contralateral eyes (median [IQR], 85.92% [69.82% to 93.98%]) (Figure 3). It should be noted that the relative thinning of the RNFL (*P* = 0.4984) and GCC (*P* = 0.8736) in the injured eyes was not significantly related to the difference in spherical equivalent (SE) between the injured and contralateral eyes (Supplemental Figure 4), suggesting that refractive error did not affect the observed thinning.

Here, we enlisted visual functional parameters and inner retinal thickness as potential noninvasive biomarkers for assessing the severity of neurodegenerative diseases. Since TON shows progressive neurodegeneration, the time interval between injury and testing was also identified as a potential neurodegenerative biomarker (19). Longitudinal data from 48 patients with TON demonstrated progressive thinning of GCC thickness over time (Supplemental Figure 5). We analyzed the correlations among these potential biomarkers and found that the structural parameters were poorly associated with the functional and temporal parameters. For example, the *R*^2^ (*P*) values for the correlation of VA with GCC thickness and RNFL thickness were 0.06 (0.0003) and 0.04 (<0.0001), respectively (Supplemental Figure 6).

### Inner retinal thickness is strongly correlated with RGC somata density and axon densities in TON large animal models.

We then tested the correlations between these noninvasive biomarkers and histological neurodegenerative benchmarks in a clinically relevant TON goat model of optic canal injury, which had clinically similar spatiotemporal pattern of progressive retrograde neurodegeneration (19). Since VA, P-VEP, and VF tests were not available in the goat model, we utilized amplitudes of pattern electroretinogram (P-ERG) with different spatial frequencies and amplitudes of flash VEP (F-VEP) under different light intensities as alternative measures of visual function parameters.

**Figure 4:**
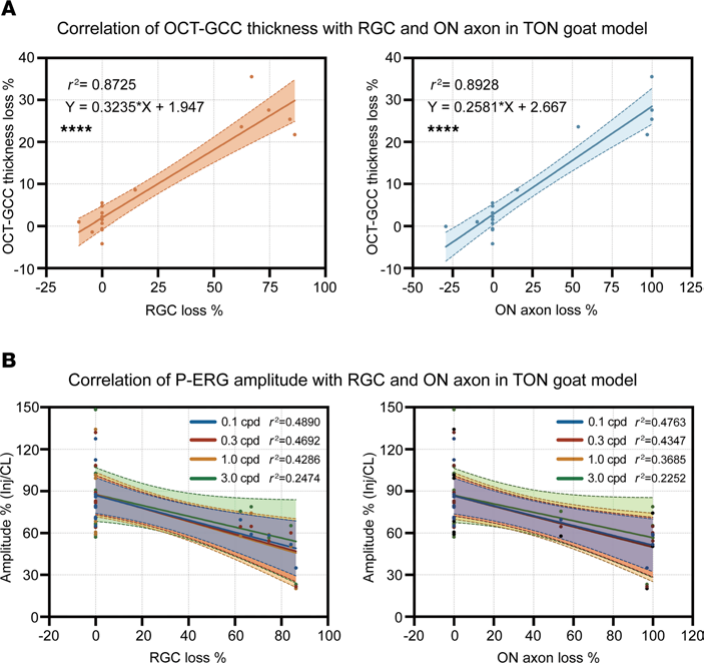
Correlation of inner retinal thickness and P-ERG amplitudes with RGC somata and ON axon densities in TON goat models. (**A**) Linear correlation of GCC thickness loss % with RGC somata (left panel) and ON axon loss % (right panel) at the primary injury site (*n* = 17 goats). (**B**) Linear correlations of P-ERG P1-N1 amplitude % (Inj/CL) at different spatial frequencies with RGC somata (left panel) and ON axon loss % (right panel) at the primary injury site (*n* = 14 goats). GCC thickness loss % = (GCC thickness of the contralateral eyes – GCC thickness of the injured eyes)/GCC thickness of the contralateral eyes. RGC loss % = (RGC somata density of the contralateral eyes – RGC somata density of the injured eyes)/RGC somata density of the contralateral eyes. ON axon loss % = (ON axon density of the contralateral eyes – ON axon density of the injured eyes at the site of injury)/ON axon density of the contralateral eyes. P-ERG P1-N1 amplitude % = P1-N1 amplitude of the injured eyes/P1-N1 amplitude of the contralateral eyes. ****P < 0.0001 by simple linear regression for **A** and **B**. RGC, retinal ganglion cell; P-ERG, pattern electroretinogram; ON, optic nerve; OCT, optical coherence tomography; GCC, ganglion cell complex.

In the TON goat model, GCC thickness loss percentage was highly correlated with both RGC somata and ON axon loss percentage on histology. The *R*^2^ (*P*) values for the correlation of GCC thickness with RGC somata loss percentage and axon loss percentage were 0.87 (<0.0001) and 0.89 (<0.0001), respectively (Figure 4A). On the other hand, measures of visual function, such as P-ERG and F-VEP amplitudes, showed lower correlations with the same histological measures. For example, the *R*^2^ (*P*) values for the correlation of P1-N1 amplitudes of P-ERG at 0.1 cycle per degree (cpd) between RGC somata loss percentage and axon loss percentage were 0.49 (0.0054) and 0.48 (0.0063), respectively (Figure 4B). The *R*^2^ (*P*) values for the correlation of P1-N1 amplitudes in F-VEP at 0.025 d.s/m^2^ with RGC somata loss percentage and axon loss percentage were 0.46 (0.0028) and 0.39 (0.0078), respectively. The time interval from the onset of TON injury to histological assessment was also moderately correlated with RGC somata and axon densities. The *R*^2^ (*P*) values for the correlation of the time interval with RGC somata loss percentage and axon loss percentage were 0.42 (0.0012) and 0.38 (0.0024), respectively (Supplemental Figure 7).

In the TON rhesus macaque model, GCC thickness also demonstrated stronger correlations with RGC somata density and axon densities compared with visual function parameters, such as P-VEP and P-ERG amplitudes at different spatial frequencies. The *R*^2^ (*P*) values for the correlation of GCC thickness with RGC somata loss percentage and axon loss percentage were 0.99 (<0.0001) and 0.95 (0.0002), respectively. In contrast, the *R*^2^ (*P*) values for the correlation between P-VEP amplitude at 0.5/1.0 d.s/m^2^ P1-N1 and RGC somata loss percentage were 0.88 (0.0019)/0.65 (0.0275), respectively. Similarly, the *R*^2^ (*P*) values for the correlation between P-ERG amplitude at 0.1, 0.3, and 1.0 cpd and RGC somata loss percentage were 0.76 (0.0104), 0.58 (0.0455), and 0.46 (0.0917) (Supplemental Figure 8).

Therefore, the inner retinal thickness, not visual functions or time interval, could be employed as a sensitive neurodegenerative biomarker because of its stronger correlation with neurodegenerative benchmarks.

### Utilizing GCC thickness loss to stratify TON severity substantially enhances clinical power.

**Figure 5:**
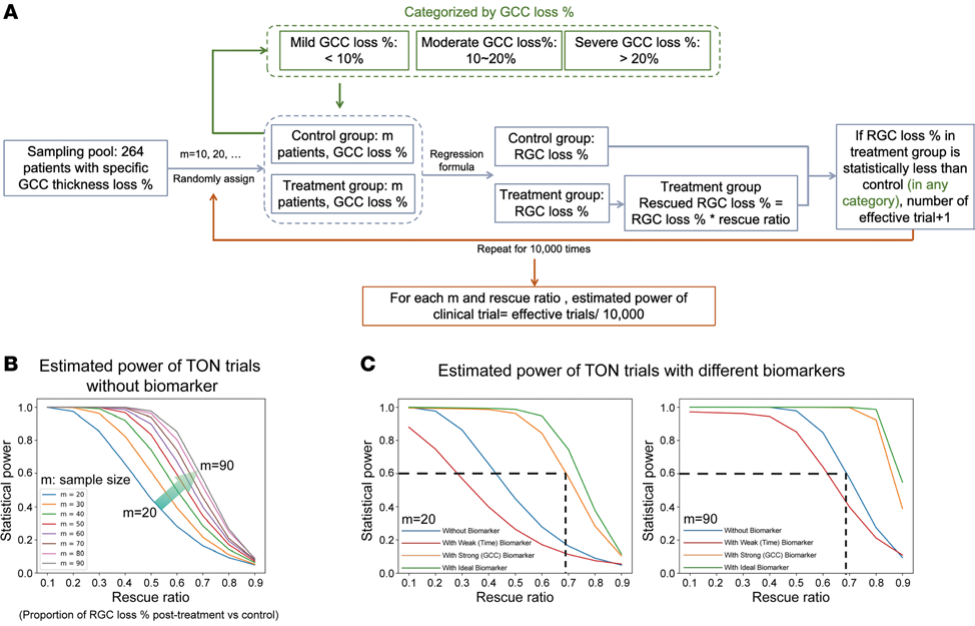
Computer simulations demonstrating that stratification of TON severity using GCC thickness enhances the statistical power of clinical trials. (**A**) Flowchart of the computational modeling. (**B**) Estimated powers of clinical trials with different sample sizes (m values) and treatment effects (larger rescue ratios indicate poorer therapeutic effect sizes) in the absence of biomarkers. (**C**) Distribution of estimated powers of TON clinical trials without biomarker (blue curve), with weak (time) biomarker (red curve), with strong (GCC) biomarker (orange curve), and with ideal biomarker (green curve). Refer to the *Computational Modeling* section in the Methods for specific statistical methods. GCC, ganglion cell complex.

The aforementioned results identified GCC thickness as a reliable neurodegenerative biomarker in TON, suggesting that incorporating GCC thickness to stratify neurodegenerative severity in TON clinical trials could enhance statistical power and consequently reduce the required sample size. To estimate the effect of GCC thickness on clinical trial efficiency, we developed a computational model (Figure 5A). The RGC somata loss percentage was calculated by comparing the RGC somata density in the injured eye with that in the contralateral healthy eye. The rescue ratio represents the proportion of RGC somata loss percentage rescued in the treated group, with smaller ratios indicating larger therapeutic effect sizes. The in silico analyses revealed several key findings.

As expected, the estimated statistical power of clinical trials without a biomarker increased with a larger sample size (m) and decreased with a poorer therapeutic effect size (indicated by a larger rescue ratio) (Figure 5B and Supplemental Figure 9).

When TON severity was stratified using an ideal biomarker (which had a perfect correlation with RGC somata loss percentage, *R*² = 1), the statistical power of clinical trials was higher than that of trials conducted without a biomarker, especially for those with moderate or mild therapeutic effect sizes (i.e., rescue ratios between 0.6 and 0.8) (Figure 5C and Supplemental Figure 9).

When TON severity was stratified using GCC thickness loss percentage, a biomarker strongly correlated with RGC somata loss percentage (*R*² = 0.87), the statistical power of clinical trials increased substantially compared with trials without a biomarker, though the increase was slightly less than that with the perfect biomarker (Figure 5C and Supplemental Figure 9). This substantial increase in statistical power indicated that a much smaller sample size was required for effective clinical trials. For example, with a target statistical power of 0.6 and a rescue ratio of approximately 0.7, a sample size of 90 patients per group would be necessary without a biomarker. In contrast, the required sample size dropped dramatically to 20 patients per group when using GCC thickness as a biomarker to stratify patients, representing a substantial 4.5-fold decrease, highlighting the powerful impact of biomarker usage (Figure 5C).

Conversely, stratifying TON severity with a weak biomarker that mildly correlated with RGC somata loss (*R*² = 0.4), such as time interval or visual function parameters, the statistical power of clinical trials decreased compared with trials without a biomarker, possibly because of the smaller sample size resulting from stratification (Figure 5C).

**Figure 6:**
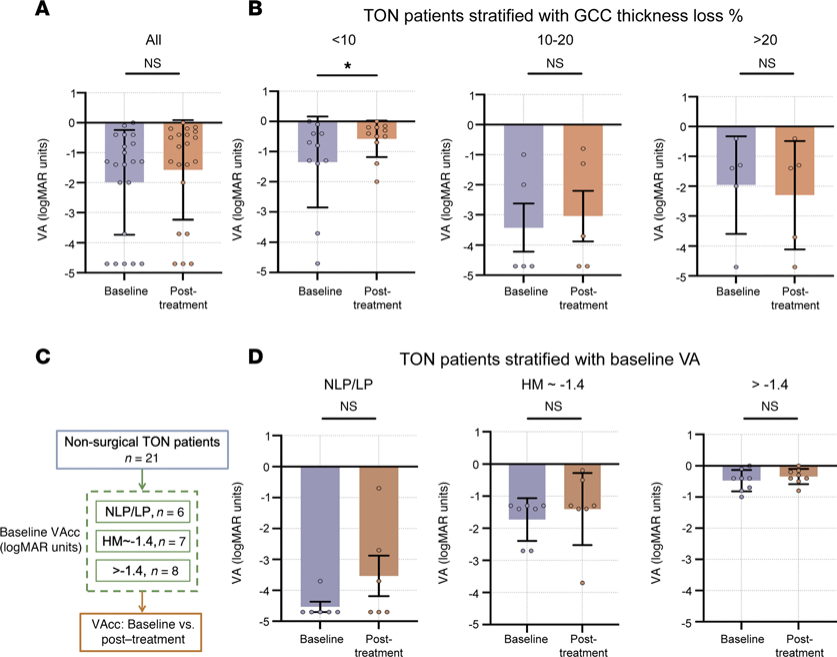
Validation of computational model findings by real-world data. (**A**) Comparison of VAs before and after treatment in patients with TON without stratification (*n* = 21 in total). (**B**) Comparison of VAs before and after treatment in patients with TON stratified by baseline GCC thickness loss percentage (<10%, *n* = 11; 10%–20%, *n* = 5; >20%, *n* = 5). (**C**) Flowchart of TON patients’ stratification with baseline logMAR VA. (**D**) Comparison of VAs before and after treatment in patients with TON stratified by baseline VA (NLP/LP, *n* = 6; HM ~–1.4, *n* = 7; >–1.4, *n* = 8; all, *n* = 21). *P < 0.05, **P < 0.01 by paired 2-tailed *t* test for **A**, **B**, and **D**. Data represent mean ± SEM. VAcc, corrected visual acuity; VA, visual acuity; NLP, no light perception; MAR, minimum angle of resolution; LP, light perception; HM, hand motion; GCC, ganglion cell complex.

To validate our computational model, we analyzed real-world data from a cohort of 21 patients with unilateral TON who had received pharmaceutical treatment. The detailed demographic and clinical characteristics of these patients are provided in Supplemental Table 3. Initial analysis of the entire cohort without stratification revealed no substantial improvement in VA at 2–3 weeks posttreatment compared with the baseline (Figure 6A). However, when we stratified patients using GCC thickness loss as a neurodegenerative biomarker, the subgroup with GCC thickness loss of less than 10% exhibited statistically significant improvement in VA following treatment (*P* < 0.01, Figure 6B). In contrast, stratification based on baseline VA, a parameter with weak correlation to neurodegeneration, failed to identify any subgroups with notable posttreatment VA improvement (Figure 6, C and D). Additionally, treated eyes in patients with GCC loss of less than 10% demonstrated significant recovery in VFI (*P* = 0.0039) and MD (*P* = 0.0195) (Supplemental Figure 10). These clinical data corroborate our computational model and support the use of GCC thickness as a robust neurodegenerative biomarker for patient stratification in TON clinical trials.

### Factors associated with inner retinal thickness loss in patients with TON.

**Figure 7:**
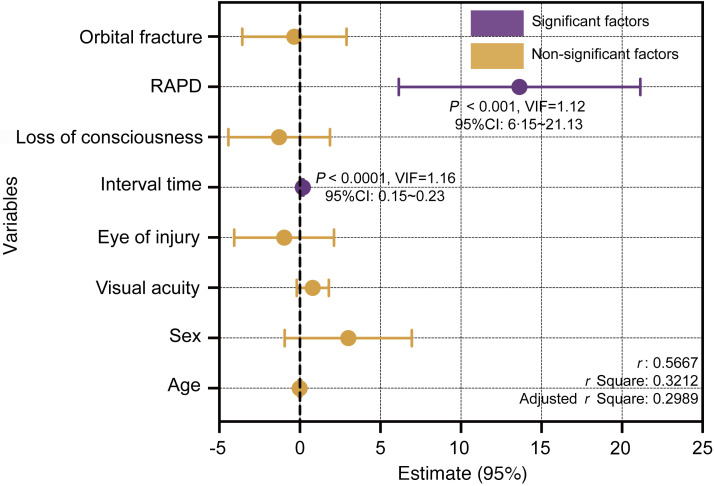
Factors associated with GCC thickness loss in TON. The *x* axis represents the beta values (β) from the multiple linear regression analysis (*n* = 254), which indicate the size and direction of each variable’s effect. Statistical significance was considered at *P* < 0.05 by multiple linear regression. VIF, variance inflation factor; RAPD, relative afferent pupillary defect.

Multiple linear regression analysis revealed that GCC thickness loss was significantly associated with the time interval between injury ophthalmic examination (β = 0.19, 95% CI, 0.15–0.23, *P* < 0.0001) and RAPD positivity (β = 13.64, 95% CI, 6.15–21.13, *P* < 0.001) and was not significantly associated with VA, age, sex, the eye of injury, history of coma, and the presence of orbital fracture (Figure 7).

### Progressive GCC thinning observed in several ON diseases.

Previous studies reported that GCC thickness was better than functional measures, such as VA and VF tests, in quantifying disease progression of glaucoma (20–22). Here we analyzed longitudinal data from 25 randomly chosen patients with primary open angle glaucoma (POAG) at our eye hospital and found similar results (Supplemental Figure 11). Both VFI (*P* = 0.0124) and MD (*P* = 0.007) in VF testing, as well as GCC (*P* < 0.0001), declined significantly between 2 randomly chosen follow-ups based on the paired 2-tailed *t* test for these patients. Interestingly, while GCC thinning was observed in 100% of the 25 patients between follow-ups, only 68% and 84% of patients exhibited worsening VFI and MD, respectively. To further explore this, we hypothesized that when pooling smaller subsets of these 25 patients, GCC would consistently demonstrate statistically significant progression between follow-ups, whereas VFI and MD might not. We found that the progression detection rate of GCC was significantly higher than those of VFI and MD for subsets with sample size between 5 and 20 (for subsets with *n* ≥ 21, progression detection rates reached 100% for all these parameters), indicating that GCC thickness was more sensitive in detecting glaucoma progression than MD and VFI in this cohort.

We further analyzed a cohort of 45 patients with anterior ischemic optic neuropathy (AION) at our hospital and found GCC thickness initially showed a transient increase during the early stages due to optic disc edema, followed by a rapid decline. In contrast, their VF parameters, such as VFI (*P* = 0.0103) and MD (*P* = 0.0192), exhibited partial recovery between the baseline and follow-up (Supplemental Figure 12). These findings were consistent with previous studies (23–25), indicating that progressive neurodegeneration in AION, characterized by axonal and somatic degeneration, may be more effectively reflected by structural measures like GCC thickness than by functional assessments such as VA or VF testing.

## Discussion

OCT is a noninvasive imaging technique that provides high-resolution retinal images, allowing precise measurements of retinal layers, such as RNFL and GCC thickness, which reflect structural neurodegenerative changes (26). Pupillometry, including RAPD assessment, evaluates the functional integrity of the PLR pathway. Combined with VA and VF testing, these ophthalmological measures theoretically provide a comprehensive assessment of both structural and functional deficits in optic neuropathies.

Clinically relevant large animal models are essential for validating noninvasive neurodegenerative biomarkers. Our model is the first to our knowledge to replicate TON in humans by incorporating optic canal injury (19). Unlike retrobulbar or retinal crush models, which lack clinical relevance, as TON typically originates in the optic canal (27–29), our model provides an accurate and translatable framework that reflects the spatiotemporal progression of TON. TON undergoes progressive retrograde neurodegeneration during disease progression (19, 30, 31): (a) short-distance axon disconnection at the optic canal, (b) long-distance axon disconnection over weeks, and (c) death of RGC somata starting approximately 1 month postinjury (Supplemental Figure 1). While the functional deficits of individual RGCs may appear similar across these stages, the structural neurodegenerative severity, quantified by RGC somata and axon loss, varies substantially. As a result, visual function, though critical for assessing the degree of recovery after treatment, does not fully reflect the extent of the structural injury prior to intervention. Our findings highlight the importance of stratifying patients in TON clinical trials based on their stage of neurodegeneration before initiating treatment.

While our findings suggest that GCC thickness may serve as a biomarker to stratify neurodegenerative severities in other optic neuropathies, such as glaucoma and ischemic optic neuropathy — both characterized by progressive degeneration of RGC somata and axonal loss — separate validation is necessary before extending its application to these nontraumatic etiologies. This validation should involve longitudinal, comparative studies in clinically relevant large animal models with histological measurement or direct measurement of RGC somata and axon densities in patients using advanced noninvasive imaging techniques, such as adaptive optics or in vivo RGC labeling (32, 33).

In addition to retinal OCT, diffusion tensor imaging (DTI) and other magnetic resonance imaging (MRI) techniques are also sensitive methods for evaluating the severity of neurodegeneration in TON (34, 35). For example, DTI-derived axial diffusivity decreases when axonal degeneration occurs, and radial diffusivity increases during demyelination (34, 35). However, several challenges limit the use of MRI in assessing the severity of TON neurodegeneration. First, MRI machines are substantially more expensive than retinal OCT devices, costing approximately 4 million USD compared with 0.14 million USD. Moreover, MRI scans are substantially more time-consuming than retinal OCT imaging, taking approximately 30 minutes as opposed to just 1 minute. Consequently, MRI scans are costlier (approximately 100 USD compared with 20 USD) and more challenging to schedule (requiring a wait of several days vs. a few hours). Furthermore, the spatial resolution of clinically available MRI (approximately 300 μm at 3.0 T) is considerably lower than that of retinal OCT (1 μm) (36). Additionally, analyzing MRI-DTI data necessitates complex processing and often includes manual artifacts.

Recent insights into ON-regenerative medicine emphasize that careful patient selection is crucial for the success of initial therapeutic interventions (37). While our biomarker approach enhances the statistical power of clinical trials, the selection of an appropriate patient population with specific disease characteristics and temporal parameters may prove equally important for demonstrating treatment efficacy. Our enhanced trial design framework, combined with strategic patient selection considerations, could substantially advance the field’s ability to identify effective neuroprotective and regenerative strategies.

In summary, this study reports the epidemiology and clinical features of indirect TON in 1,226 patients selected from 15 clinical eye centers across mainland China from 2017 to 2024. To the best of our knowledge, this is the largest multicenter nationwide epidemiological survey of patients with TON. To identify reliable biomarkers for assessing the severity of neurodegeneration, we conducted a combined analysis of clinical data from patients with monocular TON and laboratory data from large animal models that mimic clinical TON, specifically goats and rhesus macaques with optic canal injuries. We found that inner retinal thickness, rather than visual function, had a strong and significant correlation with histological RGC somata and axon loss (Figure 4). Computer modeling analysis revealed that using inner retinal thickness as a biomarker to stratify TON severity could increase the power of TON clinical trials by up to 4-fold and reduce the sample size by up to 75% (equivalent to a 4-fold decrease). This finding is crucial for managing rare conditions like TON, enabling quicker and more resource-efficient trials, thereby accelerating the development and approval of effective treatments.

### Limitations of this study.

The inner retina may undergo transient thickening within 1–2 weeks after injury (30), probably due to inner retinal edema induced by the neuroinflammatory response. Future studies are needed to screen for neurodegenerative biomarkers in the early stages of TON. Additionally, the pharmaceutical treatments evaluated in this study, including high-dose methylprednisolone and nerve growth factor, lack robust evidence of efficacy and require further studies to confirm their therapeutic potential in TON (17, 38). Furthermore, while our computer simulation analysis of 25 randomly selected patients with POAG showed significantly higher progression detection rates for GCC compared with VFI and MD for sample sizes between 5 and 20 (Supplemental Figure 11E), we acknowledge that this observation is limited by the small cohort size and should be validated in larger, multicenter studies with longer follow-up periods. While our simulation-based stratification approach demonstrates theoretical enhancement of statistical power, we acknowledge that prospective validation in future clinical trials would provide more definitive evidence of GCC thickness’s utility as a stratification biomarker.

## Methods

### Sex as a biological variable.

Both male and female patients with TON were included in this study, but sex was not considered as a biological variable in the analysis. All animal experiments were performed using male Saanen goats and rhesus macaques, as the majority of clinical TON patients were men.

### National Multi-Center Collaborative Clinical Research Program for TON.

The Chinese National Multi-Center Collaborative Clinical Research Program for TON was established in 2017. This prospective study was conducted at multiple clinical eye centers that enrolled patients with newly diagnosed TON and collected relevant clinical data using a networked digital system for centralized analysis. From November 2017 to November 2024, 1,226 patients with TON were enrolled from 15 hospitals in 10 provinces of China (Figure 1A). This study followed the Strengthening the Reporting of Observational Studies in Epidemiology reporting guidelines (39).

TON was diagnosed based on the occurrence of severe VA or VF loss following a recent closed head trauma (occurring within 2 weeks of ocular evaluation and diagnosis) with or without an RAPD. Patients in whom visual loss was attributed to global trauma or intracranial injury were excluded (10, 27). Binocular TON was diagnosed when both eyes of a patient demonstrated severe visual loss following a recent closed head trauma that was not explained by other causes. Patients who were unconscious or unable to communicate during hospitalization at clinical eye centers were excluded.

### Clinical data collection and analysis.

This multicenter prospective observational study collected the epidemiological and clinical data from all 1,226 patients with TON during their hospitalization on the day of admission. Most patients underwent ophthalmic examinations, including patient history, VA, RAPD test, slit lamp biomicroscopy, and intraocular pressure measurement. Some of them received additional, more comprehensive examinations, such as OCT of the optic disc, VF examination using automated perimetry, and P-VEP. The decision to administer these additional tests was primarily based on the doctor’s advice or the availability of the tests rather than any strict criteria.

OCT scans of the optic disc were obtained using a spectral OCT machine (Optovue) at WMU Eye Hospital. The average RNFL thickness was measured using an automated segmentation algorithm (30), whereas GCC thickness was manually measured and calibrated by 2 expert ophthalmologists. The percentage of RNFL or GCC thickness loss was defined as the ratio of the reduced thickness in the study eye experiencing vision loss (Ti-Tc) to the thickness in the contralateral unaffected eye (Tc) in patients with monocular TON. To exclude the effect of myopia on inner retinal thinning, we analyzed the correlation between the percentage of RNFL and GCC thickness loss with SE in injured eyes (40). P-VEP recordings were performed using the GT-2008V-III system (GOTEC Co., Ltd). The amplitude and the latency of P100 wave, with a latency of approximately 100 ms, were compared between the injured and the contralateral healthy eyes (41). VF testing was conducted using Humphrey automated perimeter and the 30–2 program using the Swedish Interactive Threshold Algorithm standard. The VFI percentage and MD were compared between the injured and contralateral eyes (42).

### Preclinical large animal models of TON.

The study involved juvenile male Saanen goats (*Capra aegagrus hircus*), aged between 4 and 7 months (considered young adults for goats) and weighing 19–22 kg, which were sourced from the Caimu Livestock Company in Hangzhou, China, and housed at WMU’s animal facility. Additionally, adult rhesus macaques (*Macaca mulatta*) aged 5–7 years (considered young adults for rhesus macaques), weighing 5–7 kg, were maintained at the Joinn Laboratory’s animal facility.

The retrobulbar ON crush model has been widely used in laboratory research to study TON. However, most clinical TON cases occur in the optic canal (27). Recently, we established a clinically relevant model of TON in large animal models by inducing nerve injury in the optic canal, as previously described. The data from the large animal model used in this study were derived from our previous study (19). The methods for RGC counts, including the preparation of retinal sections, staining, and quantification, were performed as described in our previous study. Briefly, we exposed the left prechiasmatic ON of the large animal and applied a crush injury using microinvasive transnasal endoscopy. F-VEP, and P-ERG (which reflects RGC functions and retinal spatial resolution) (43) were conducted at 1 day, 1 week, 1 month, and 3 months postinjury. Histological studies to quantify RGC somata and axon densities were performed in both the injured and contralateral control eyes before and after crush injury at 1 month and 3 months. To reflect the wide range of neurodegenerative severities observed in clinical TON, longitudinal data from the sham group were also included in our single linear regression analysis between noninvasive biomarkers and histological benchmarks. In the sham treatment group, the intracanalicular ON was exposed without performing a crush injury.

### Computational modeling.

This study employed computational modeling to estimate the potential increase in the statistical power of clinical trials by using neurodegenerative biomarkers to stratify neurodegenerative severity. The statistical power of clinical trials depends on the sample size, effect size, and data distribution: A narrower data distribution enhances the statistical power for a given sample size and effect size. In this study, we categorized GCC thickness loss into 3 levels: mild (<10%), moderate (10%–20%), and severe (>20%), ensuring an approximately equal sample size in each category in this study.

Data from the National Multi-Center Collaborative Clinical Research Program included 264 patients with monocular TON who underwent bilateral GCC measurements on their admission day. The percentages of GCC thickness loss in the injured eyes, compared with the contralateral healthy eyes, were recorded and used as the sampling pool. We randomly assigned m (m = 20, 30, 40..., 90) patients from the total of 264 patients to both the control and treatment groups. Their GCC thickness loss percentages were converted to RGC somata loss percentages using a regression equation derived from the TON large animal model (RGC somata loss % = 2.697 × GCC thickness loss % – 2.445, 95% CI, Slope: 2.130 to 3.265, Y-intercept: –11.00 to 6.110) (44).


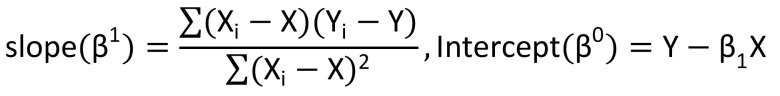


Due to *R*² < 1, each GCC thickness loss percentage corresponded to a range of RGC somata loss percentage. This range was determined by the 95% CI for the slope and intercept. The probability density of each predicted RGC somata loss value within this range was obtained using the probability density function of the normal distribution, as follows:


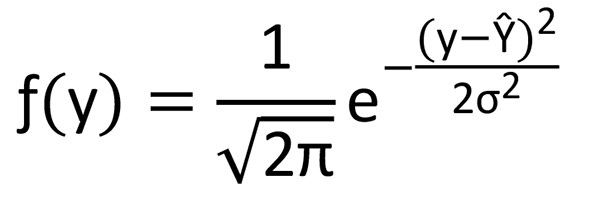


(σ: standard error; the further a predicted Ŷ value is from the expected value, the lower its probability density) (45).

In the treatment group, each RGC somata loss percentage was presumably rescued by a rescue ratio (ranging from 0.1 to 0.9, with smaller ratios indicating larger therapeutic effect sizes). We compared the final percentages of RGC somata loss between the treatment and control groups using an unpaired 2-tailed *t* test or Wilcoxon rank-sum test depending on the normality of the data. If the RGC somata loss percentage in the treatment group was statistically lower than that in the control group, we considered the sampling round an effective clinical trial. This sampling process was repeated 10,000 times, and trial power was calculated as the proportion of effective trials out of 10,000.

For computational modeling of patient stratification, the overall procedure was quite similar, except for 3 key differences. First, the randomly assigned m patients were categorized according to the severity of GCC thickness loss (mild, moderate, and severe) in both the treatment and control groups. Next, comparisons were made between the treatment and control groups within each category. Finally, if the RGC somata loss percentage was significantly lower in the treatment group within any category, the round was deemed an effective trial.

### Confirmation of the computational findings using real-world data.

Computational analysis suggested that stratifying patients with TON based on GCC thickness loss could significantly enhance the power of clinical trials. To test this finding preliminarily, we analyzed data from 21 patients with monocular TON, who were selected unbiasedly from our database. These patients underwent a 3-day course of 500 mg methylprednisolone combined with mouse nerve growth factor treatment at the Eye Hospital, WMU. They subsequently underwent follow-up eye examinations 2–3 weeks posttreatment. VAcc was measured after the treatment and compared with the baseline VA, with or without stratification.

### Longitudinal assessment and progression analysis of patients with TON, POAG, and AION.

In our database, there were 48 patients with unilateral TON who underwent 2 follow-up visits and received complete opthalmologic examinations, including OCT and VF analysis. We analyzed longitudinal data of these patients. VA, OCT-derived GCC thickness, MD, and VFI were measured during follow-up assessments at Wenzhou Eye Hospital.

Longitudinal data from 25 randomly selected patients with POAG and 45 randomly selected patients with AION were analyzed. In both groups, OCT-derived GCC thickness, VFI, MD, and VA were measured between follow-ups at Wenzhou Eye Hospital from October 2024 to January 2025. The GCC thickness was adjusted for normal age-related thinning (–0.2%/year) (46).

POAG was diagnosed based on characteristic glaucomatous damage to the ON head and RNFL, confirmed by fundoscopic examination, OCT, and glaucomatous VF defects through automated perimetry, with an open anterior chamber angle verified by gonioscopy. Patients with elevated or normal intraocular pressure were included, while other ON disorders, such as hereditary or compressive optic neuropathies, were excluded (47–49).

AION was diagnosed in patients with sudden, painless vision loss and/or VF defects, typically showing quadrant defects connected to the physiological blind spot, often in the nasal or inferior fields. Fundoscopic examination revealed ON head edema, often with peripapillary hemorrhages. RAPD and abnormalities in VEP were also noted. Patients with other ON disorders were excluded (50, 51).

We also developed a computer program for POAG to calculate the progression detection rates of GCC, VFI, and MD in each pooled subset (*n* = 5–20). Briefly, we randomly selected subsets of *n* patients at both the baseline and first follow-up and tested whether their GCC, VFI, and MD values showed a statistically significant decline between the 2 time points. Before statistical testing, normality was assessed for each subset. For normally distributed data, paired 2-tailed *t* tests were used, whereas non-normally distributed data were analyzed using the Wilcoxon signed-rank test. This process was repeated 1,000 times for each *n*. The progression detection rate was defined as the proportion of samplings showing a statistically significant decline out of 1,000 repetitions.

### Statistics.

Statistical analyses and graph plotting were performed using GraphPad Prism, version 8.0.2 (GraphPad Software), and ArcGIS Desktop (ESRI), version 10.8. *P* < 0.05 was considered statistically significant. The Shapiro-Wilk test was used to test the normality of the data. The χ^2^ test was used to analyze categorical variables. The 2-tailed *t* test was used for analysis of continuous variables with normal distribution, whereas the Wilcoxon rank-sum test was used for analysis of continuous variables without normal distribution. Simple linear regression was used to explore the correlation between 2 continuous variables. Multiple linear analysis was used to screen for factors that may be correlated with the interval between injury and hospitalization and RNFL loss. While *R*² values were used to report the strength of the correlations, the directional nature (positive or negative) of these correlations was illustrated in the figures. McNemar’s and Fisher’s exact test was used to compare the detection rates in subsets with the same number of patients.

### Study approval.

The TON study was approved by the ethical committee of the Affiliated Eye Hospital of WMU (Y2017-057) and registered in the Chinese Clinical Trial Registry (ChiCTR-OOC-17013437). The POAG (2024-179-K-149-01) and AION (2025-032-K-028) studies were approved by the same ethical committee. Written informed consent was obtained from all participants prior to their inclusion in the study. For any photographic material used, specific written informed consent was secured.

All animal experiments adhered to the guidelines set by the Association for Research in Vision and Ophthalmology concerning the use of animals in vision and ophthalmic research. All experimental procedures received approval from the Institutional Animal Care and Use Committees of Wenzhou Medical University (wydw2020-0789) and at the Joinn Laboratory (P19-S445-PD), located in Wenzhou and Suzhou, China, respectively.

### Data availability.

Data used in this study can be obtained upon a reasonable request from the corresponding author. All underlying data for the figures and tables in this article have been compiled into a single Excel document named Supporting Data Values. Each figure part or table has been organized for easy reference. The original code used for the TON and POAG computer modeling in this article is available on GitHub via this link: https://github.com/xby-0823/Computational-modeling.git (commit ID 1b5eacf).

## Author contributions

WCW and YKZ had full access to all of the data in the study and take responsibility for the integrity of the data and the accuracy of the data analysis. YKZ and BYX contributed to the work equally and should be regarded as co–first authors. The order of the co–first authors was assigned by a mutual agreement between YKZ and BYX.

Conception and design were performed by WCW and YKZ. Acquisition, analysis, or interpretation of data were performed by YKZ, BYX, SWH, ZHS, WX, RW, GQL, LC, ZHG, YJZ, HLL, BYJ, CXW, HHS, JK, NYA, DFC, SHH, YTL, MYL, ZWW, WY, ZHY, YHT, EDW, KZ, WCW, GL, and JZ. Drafting of the manuscript was done by YKZ and BYX. Critical revision of the manuscript for important intellectual content was done by YKZ, BYX, YHT, KZ, and WCW. Statistical analysis was performed by YKZ and BYX. Computer modeling and program development were performed by YKZ, SRH, HC, and XGD. Obtaining funding was done by WCW, YKZ, and WY. Administrative, technical, or material support were given by WCW, YKZ, WY, EDW, and YHT. Supervision was done by WCW, KZ, SHW, YKZ, EDW, and YHT.

## Supplementary Material

Supplemental data

ICMJE disclosure forms

Supporting data values

## Figures and Tables

**Table 1:**
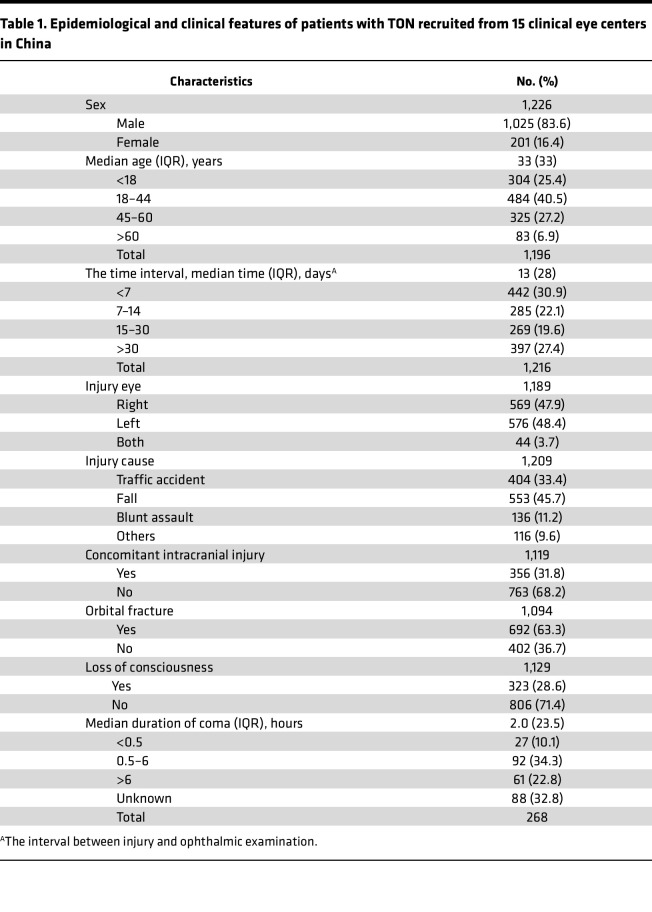
Epidemiological and clinical features of patients with TON recruited from 15 clinical eye centers in China

## Version 1 06/17/2025

In-Press Preview

## Version 2 07/22/2025

Electronic publication

## References

[1] Gise R (2018). visual pathway injuries in pediatric ocular trauma-a survey of the National Trauma Data Bank from 2008 to 2014. Pediatr Neurol.

[2] Zwerling CS (2022). Current review of traumatic optic neuropathy and traumatic brain injury among military service members. Recent Trends Pharm Sci Res.

[3] Karimi S (2021). A systematic literature review on traumatic optic neuropathy. J Ophthalmol.

[4] Yu-Wai-Man P (2015). Traumatic optic neuropathy-Clinical features and management issues. Taiwan J Ophthalmol.

[5] Crompton MR (1970). Visual lesions in closed head injury. Brain.

[6] Ford RL (2012). A 2-year prospective surveillance of pediatric traumatic optic neuropathy in the United Kingdom. J AAPOS.

[7] Levin LA (1999). The treatment of traumatic optic neuropathy: the International Optic Nerve Trauma Study. Ophthalmology.

[8] Yu B (2018). Outcome of endoscopic trans-ethmosphenoid optic canal decompression for indirect traumatic optic neuropathy in children. BMC Ophthalmol.

[9] Kashkouli MB (2018). Traumatic optic neuropathy treatment trial (TONTT): open label, phase 3, multicenter, semi-experimental trial. Graefes Arch Clin Exp Ophthalmol.

[10] Lee V (2010). Surveillance of traumatic optic neuropathy in the UK. Eye (Lond).

[11] Wei W (2022). The outcome of surgical and non-surgical treatments for traumatic optic neuropathy: a comparative study of 685 cases. Ann Transl Med.

[12] Emanuelli E (2015). Post-traumatic optic neuropathy: our surgical and medical protocol. Eur Arch Otorhinolaryngol.

[13] Carta A (2003). Visual prognosis after indirect traumatic optic neuropathy. J Neurol Neurosurg Psychiatry.

[14] Yang QT (2012). The therapeutic efficacy of endoscopic optic nerve decompression and its effects on the prognoses of 96 cases of traumatic optic neuropathy. J Trauma Acute Care Surg.

[15] Yang SG (2020). Strategies to promote long-distance optic nerve regeneration. Front Cell Neurosci.

[16] Winter CC (2022). Axon regeneration: a subcellular extension in multiple dimensions. Cold Spring Harb Perspect Biol.

[17] Wladis EJ (2021). Interventions for indirect traumatic optic neuropathy: a report by the American Academy of Ophthalmology. Ophthalmology.

[18] Singman EL (2016). Indirect traumatic optic neuropathy. Mil Med Res.

[19] Zhang Y (2022). Cold protection allows local cryotherapy in a clinical-relevant model of traumatic optic neuropathy. Elife.

[20] Kuang TM (2015). Estimating lead time gained by optical coherence tomography in detecting glaucoma before development of visual field defects. Ophthalmology.

[21] Swaminathan SS (2021). Rapid initial OCT RNFL thinning is predictive of faster visual field loss during extended follow-up in glaucoma. Am J Ophthalmol.

[22] Zhang X (2017). Comparison of glaucoma progression detection by optical coherence tomography and visual field. Am J Ophthalmol.

[23] Contreras I (2007). Follow-up of nonarteritic anterior ischemic optic neuropathy with optical coherence tomography. Ophthalmology.

[24] Rebolleda G (2013). Visual and anatomical outcomes of non-arteritic anterior ischemic optic neuropathy with high-dose systemic corticosteroids. Graefes Arch Clin Exp Ophthalmol.

[25] García-Basterra I (2020). Prospective analysis of macular and optic disc changes after non-arteritic anterior ischemic optic neuropathy. J Fr Ophtalmol.

[26] Aref AA, Budenz DL (2010). Spectral domain optical coherence tomography in the diagnosis and management of glaucoma. Ophthalmic Surg Lasers Imaging.

[27] Yan W (2017). Incidence of optic canal fracture in the traumatic optic neuropathy and its effect on the visual outcome. Br J Ophthalmol.

[28] al-Qurainy IA (1991). The characteristics of midfacial fractures and the association with ocular injury: a prospective study. Br J Oral Maxillofac Surg.

[29] Huempfner-Hierl H (2015). Blunt forehead trauma and optic canal involvement: finite element analysis of anterior skull base and orbit on causes of vision impairment. Br J Ophthalmol.

[30] Shi W (2013). Axonal loss and blood flow disturbances in the natural course of indirect traumatic optic neuropathy. Chin Med J (Engl).

[31] Miyahara T (2003). Alterations in retinal nerve fiber layer thickness following indirect traumatic optic neuropathy detected by nerve fiber analyzer, GDx-N. Am J Ophthalmol.

[32] Rossi EA (2017). Imaging individual neurons in the retinal ganglion cell layer of the living eye. Proc Natl Acad Sci U S A.

[33] Nieuwenhuis B (2023). Improving adeno-associated viral (AAV) vector-mediated transgene expression in retinal ganglion cells: comparison of five promoters. Gene Ther.

[34] Li J (2014). Time-dependent diffusion tensor changes of optic nerve in patients with indirect traumatic optic neuropathy. Acta Radiol.

[35] Mac Donald CL (2007). Detection of traumatic axonal injury with diffusion tensor imaging in a mouse model of traumatic brain injury. Exp Neurol.

[36] Bachmann R (2007). High-resolution magnetic resonance imaging (MRI) at 3.0 Tesla in the short-term follow-up of patients with proven cervical artery dissection. Invest Radiol.

[37] Bhattacharya SK (2024). Appropriate patient population for future visual system axon regeneration therapies. WIREs Mech Dis.

[38] Yu-Wai-Man P, Griffiths PG (2013). Steroids for traumatic optic neuropathy. Cochrane Database Syst Rev.

[39] Cuschieri S (2019). The STROBE guidelines. Saudi J Anaesth.

[40] Mwanza JC (2012). Rates of abnormal retinal nerve fiber layer and ganglion cell layer OCT scans in healthy myopic eyes: Cirrus versus RTVue. Ophthalmic Surg Lasers Imaging.

[41] Sharma R (2015). Visual evoked potentials: normative values and gender differences. J Clin Diagn Res.

[42] Rao HL (2013). Behavior of visual field index in advanced glaucoma. Invest Ophthalmol Vis Sci.

[43] Origlia N (2012). Visual acuity is reduced in αlpha 7 nicotinic receptor knockout mice. Invest Ophthalmol Vis Sci.

[44] Schneider A (2010). Linear regression analysis: part 14 of a series on evaluation of scientific publications. Dtsch Arztebl Int.

[46] Zhang X (2016). Longitudinal and cross-sectional analyses of Age effects on retinal nerve fiber layer and ganglion cell complex thickness by Fourier-Domain OCT. Transl Vis Sci Technol.

[47] Berdahl JP (2008). Cerebrospinal fluid pressure is decreased in primary open-angle glaucoma. Ophthalmology.

[48] Pasquale LR (2010). Anthropometric measures and their relation to incident primary open-angle glaucoma. Ophthalmology.

[49] Weinreb RN, Khaw PT (2004). Primary open-angle glaucoma. Lancet.

[50] Hayreh SS, Zimmerman MB (2008). Nonarteritic anterior ischemic optic neuropathy: clinical characteristics in diabetic patients versus nondiabetic patients. Ophthalmology.

[51] Hayreh SS, Zimmerman MB (2008). Nonarteritic anterior ischemic optic neuropathy: natural history of visual outcome. Ophthalmology.

