# Supplementary material for: Large-scale survey, animal models, and computational modeling identify histological neurodegenerative biomarkers for traumatic optic neuropathy
Source: JCI Insight. 2025 Jun 17;10(14):e190682. doi: 10.1172/jci.insight.190682 (PMC12288978; doi:10.1172/jci.insight.190682)
Supplement: Supplemental data [file jciinsight-10-190682-s235.pdf]

Supplementary Data

**Table of Contents**	**Page**
Extended Figure 1. Comparable RGC dysfunction observed across all three stages of TON progression, despite varying degrees of neurodegeneration.	2
Extended Figure 2. Estimated minimal annual incidence of TON in Wenzhou City from 2018 to 2024.	3
Extended Figure 3. Representative fundus images of the injured and contralateral eyes in patient with unilateral TON.	4
Extended Figure 4. Correlation of RNFL and GCC thickness loss with spherical equivalent difference in affected eyes.	5
Extended Figure 5. Longitudinal changes in visual function and GCC thickness in TON Patients.	6
Extended Figure 6. Weak Correlation of inner retinal thickness with functional and temporal parameters in patients with monocular TON.	7
Extended Figure 7. Correlation of visual function and post-injury time with RGC soma density and ON axon density in TON goat models.	8
Extended Figure 8. Correlation of inner retinal thickness and P-ERG/P-VEP amplitudes with RGC soma density and ON axon density in non-human primate TON models.	9
Extended Figure 9. Computational modeling of increased statistical power through TON patient stratification using ideal or strong neurodegenerative biomarkers across varying sample sizes.	10
Extended Figure 10. Comparison of visual field metrics before and after pharmaceutical treatment in patients with unilateral TON.	11
Extended Figure 11. Longitudinal changes in visual and structural parameters in POAG.	12
Extended Figure 12. Longitudinal changes in structural and functional visual parameters in AION.	13
Extended Table 1. Previous clinical trials for TON	14-19
Extended Table 2. Comparative analysis of functional and structural parameters between the injured eye and the contralateral eye in patients with unilateral TON	20
Extended Table 3. Demographic and Clinical Characteristics of Patients with Traumatic Optic Neuropathy Receiving Pharmaceutical Treatments	21

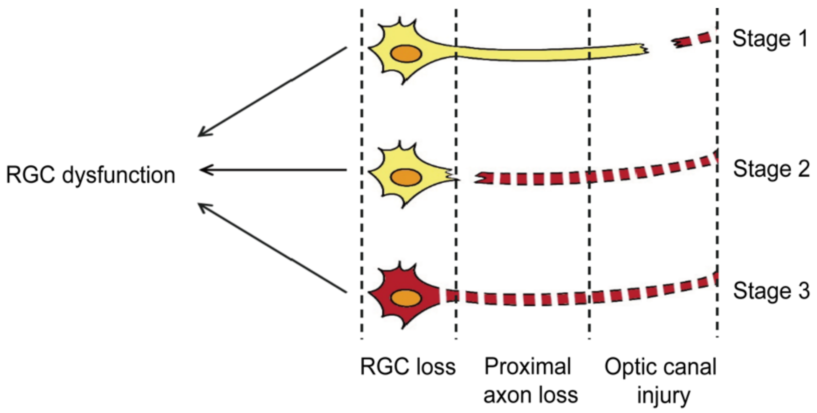

Extended Figure 1. Comparable RGC dysfunction observed across all three stages of TON progression, despite varying degrees of neurodegeneration.

The same functional deficits in individual RGCs appear across different stages: Stage 1 involves short-distance axon disconnection at the optic canal; Stage 2 is characterized by progressive retrograde long-distance axon disconnection; and Stage 3 features RGC soma death.

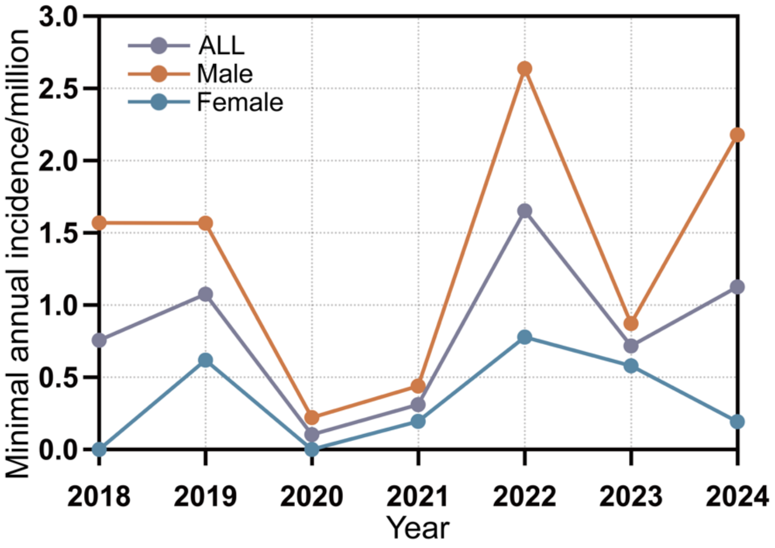

Extended Figure 2. Estimated minimal annual incidence of TON in Wenzhou City from 2018 to 2024[a].

[a] The incidence rate in 2024 is estimated based on the resident population of Wenzhou City in 2023.

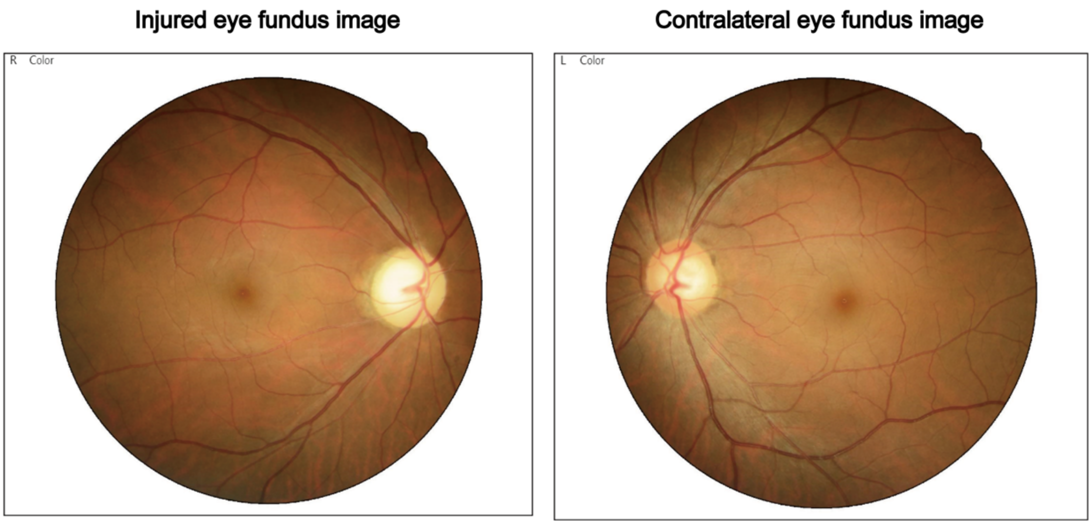

Extended Figure 3. Representative fundus images of the injured and contralateral eyes in patient with unilateral TON.

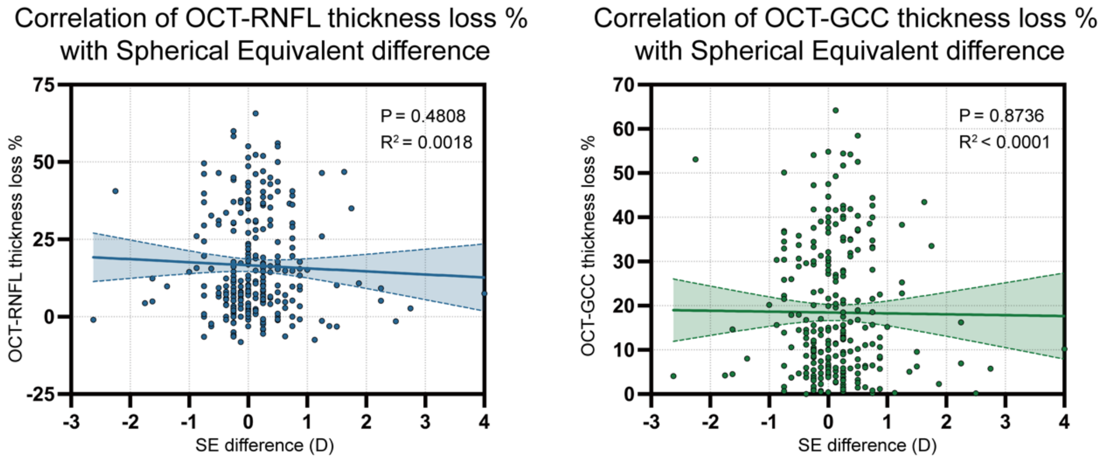

Extended Figure 4. Correlation of RNFL and GCC thickness loss with Spherical Equivalent difference in affected eyes.

Correlation between RNFL (left panel) and GCC ((right panel)) thickness loss % with SE difference (n=264). SE difference = SE of the contralateral eyes – SE of the injured eyes; Statistical analysis was performed using linear regression. ns, not significant; OCT, optical coherence tomography; RNFL, retinal nerve fiber layer; GCC, ganglion cell complex; SE, spherical equivalent.

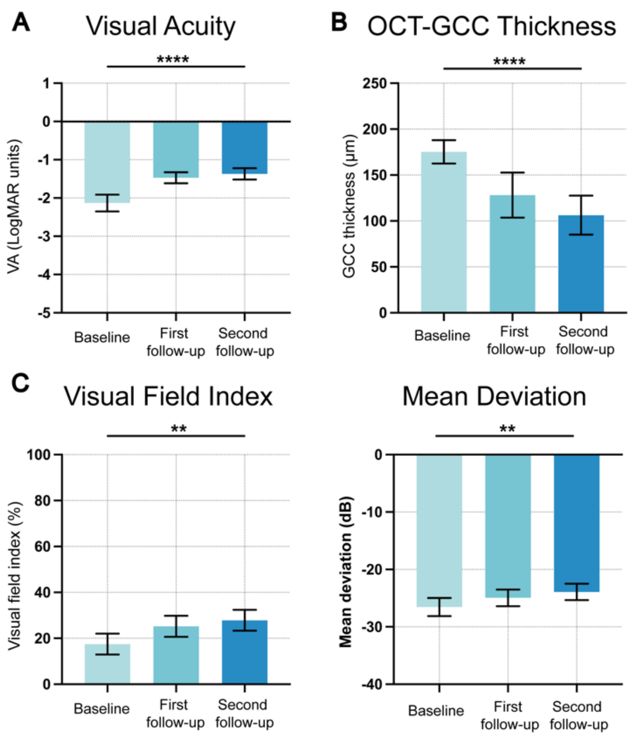

Extended Figure 5. Longitudinal changes in visual function and GCC thickness in TON Patients.

(**A**) Comparison of visual acuity at baseline, first follow-up, and second follow-up in TON patients (n=48). (**B**) Comparison of GCC thickness at baseline, first follow-up, and second follow-up in TON patients (n=45). (**C**) Comparison of VFI and MD at baseline, first follow-up, and second follow-up in TON patients (n=36). Data are presented as median (IQR). Statistical analysis was performed using One-way ANOVA test. ** $P < 0.01$, **** $P < 0.0001$. OCT, optical coherence tomography; MD, mean deviation; VFI, visual field index; TON, traumatic optic neuropathy.

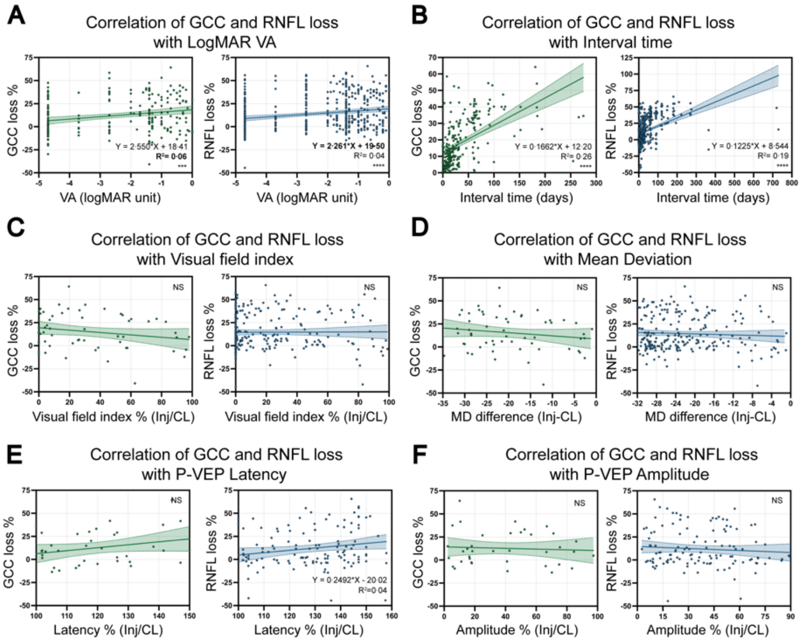

Extended Figure 6. Weak Correlation of inner retinal thickness with functional and temporal parameters in patients with monocular TON.

(**A**) Linear correlation of GCC thickness loss % (left panel, n=220) and RNFL thickness loss % (right panel, n=486) with baseline visual acuity in injured eyes. (**B**) Linear correlation of GCC thickness loss % (left panel, n=254) and RNFL thickness loss % (right panel, n=496) with interval time between injury and ophthalmic examination. (**C-D**) Linear correlation of GCC thickness loss % (left panel, n=59) and RNFL thickness loss % (right panel, n=254) with visual field index % and MD difference. (**E-F**) Linear correlation of GCC thickness loss % (left panel, n=37) and RNFL thickness loss % (right panel, n=125) with P-VEP Latency % and Amplitude %. RNFL and GCC loss % = (RNFL and GCC thickness of the contralateral eyes - RNFL and GCC thickness of the injured eyes) / RNFL and GCC thickness of the contralateral eyes. MD difference = MD of the injured eyes - MD of the contralateral eyes. VFI % = VFI of the injured eyes / VFI of the contralateral eyes. P-VEP amplitude % = P100 amplitude of the injured eyes / P100 amplitude of the contralateral eyes. P-VEP latency % = P100 latency of the injured eyes / P100 latency of the contralateral eyes.
A: ordinary one-way analysis of variance. B-C: simple linear regression.
* *P* < .05, ** *P* < .01, *** *P* < .001, **** *P* < .0001, ns: not significant.
VA, visual acuity; RNFL, retinal nerve fiber layer; MD, mean deviation; VFI, visual field index; P-VEP, pattern visual evoked potential; GCC, ganglion cell complex.

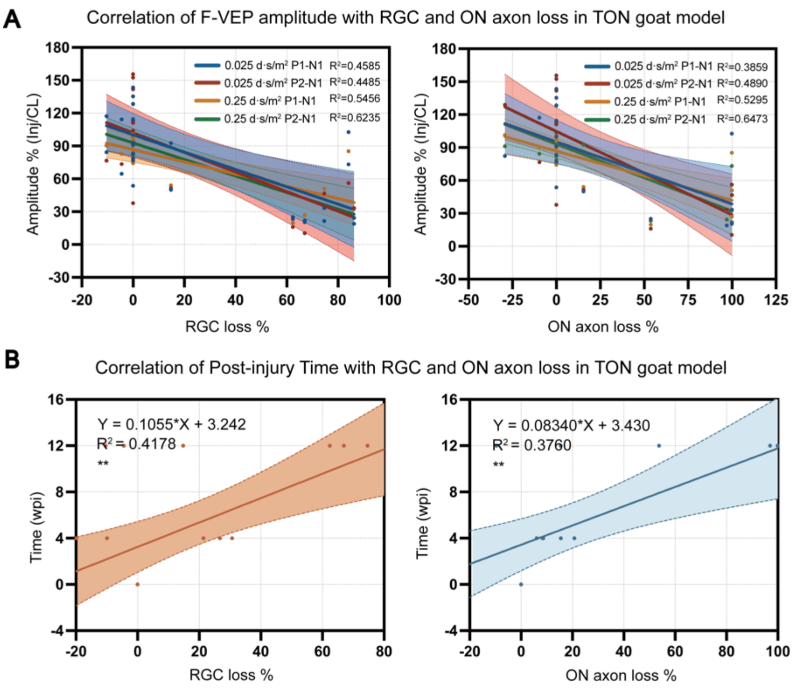

Extended Figure 7. Correlation of visual function and post-injury time with RGC soma density and ON axon density in TON goat models.

(**A**) Linear correlation of F-VEP P1/P2-N1 amplitude % at 0.025 and 0.25 cd·s/m^2 with RGC loss % and ON axon loss % (both panels n=9). (**B**) Linear correlation of post-injury time with RGC loss % and ON axon loss % (both panels n=9).

RGC loss % = (RGC density of the contralateral eyes - RGC density of the injured eyes) / RGC density of the contralateral eyes. ON axon loss % = (ON axon density of the contralateral eyes - ON axon density of the injured eyes at the site of injury) / ON axon density of the contralateral eyes. F-VEP P1/P2-N1 amplitude % = P1/P2-N1 amplitude of the injured eyes / P1/P2-N1 amplitude of the contralateral eyes.

NS, not significant; F-VEP, flash visual evoked potential; RGC, retinal ganglion cell; ON, optic nerve; cpd, cycles per degree; wpi, week post-injury.

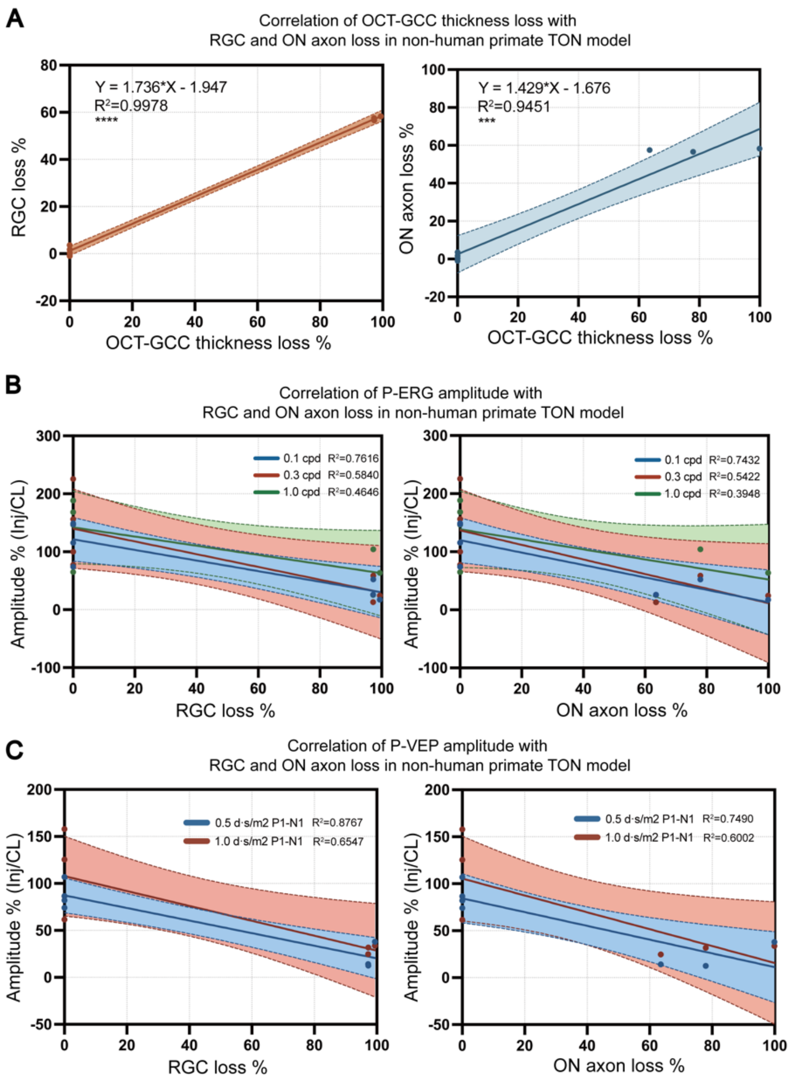

Extended Figure 8. Correlation of inner retinal thickness and P-ERG/P-VEP amplitudes with RGC soma density and ON axon density in non-human primate TON models.

(**A**) Linear correlation of GCC loss % with RGC loss % and ON axon loss % (both panels n=4). (B) Linear correlation of P-ERG P1-N1 amplitude % (spatial frequencies: 0.1, 0.3 and 1.0 cpd) with RGC loss % and ON axon loss % (both panels n=4). (**C**) Linear correlation of P-VEP P1-N1 amplitude % (spatial frequencies: 0.5 and 1.0 cd·s/m^2) with RGC loss % and ON axon loss % (both panels n=4). OCT-GCC thickness loss % = (GCC thickness of the contralateral eyes - GCC thickness of the injured eyes) / GCC thickness of the contralateral eyes. RGC loss % = (RGC density of the contralateral eyes - RGC density of the injured eyes) / RGC density of the contralateral eyes. ON axon loss % = (ON axon density of the contralateral eyes - ON axon density of the injured eyes at the site of injury) / ON axon density of the contralateral eyes. P-ERG P1-N1 amplitude % = P1-N1 amplitude of the injured eyes / P1-N1 amplitude of the contralateral eyes. P-VEP P1-N1 amplitude % = P1-N1 amplitude of the injured eyes / P1-N1 amplitude of the contralateral eyes.

OCT, optical coherence tomography; GCC, ganglion cell complex; P-ERG, pattern electroretinogram; P-VEP, pattern visual evoked potential; RGC, retinal ganglion cell; ON, optic nerve; cpd, cycles per degree.

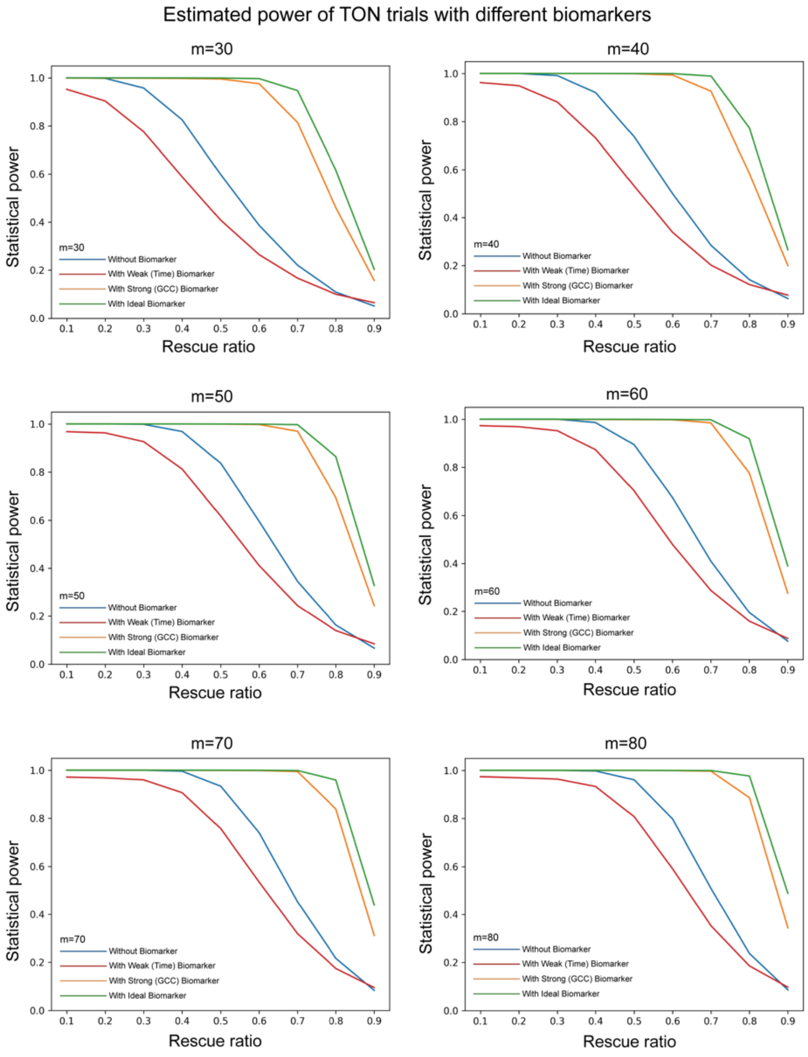

Extended Figure 9. Computational modeling of increased statistical power through TON patient stratification using ideal or strong neurodegenerative biomarkers across varying sample sizes.

Blue curve: without biomarker; Red curve: with weak biomarker (time post-injury, R^2=0.4); Orange curve: with strong biomarker (GCC, R^2=0.87); Green curve: with an ideal biomarker (R^2=1). Sample size (m) = 30, 40, 50, 60, 70, 80, 90.

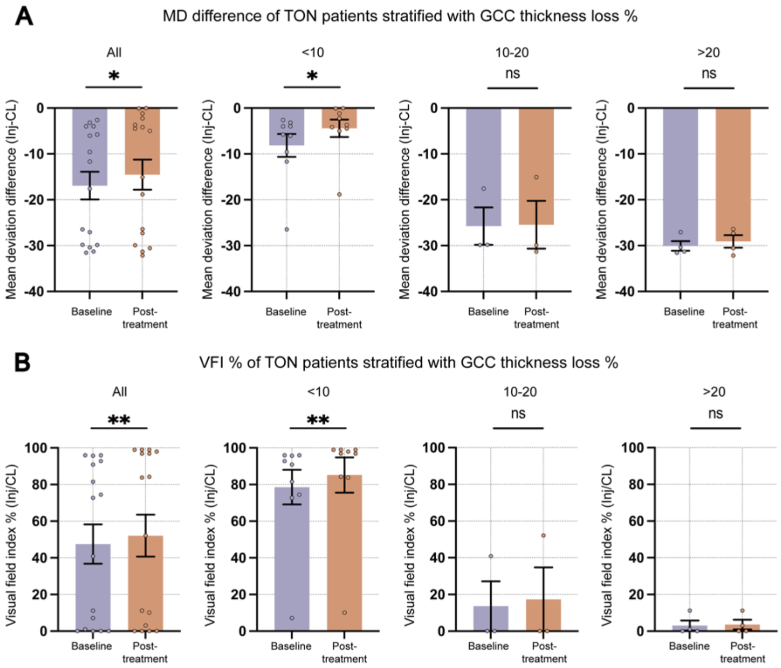

Extended Figure 10. Comparison of visual field metrics before and after pharmaceutical treatment in patients with unilateral TON.

(**A and B**) Comparison of MD difference and VFI % before and after treatment in TON patients stratified by baseline GCC thickness loss % (<10%, n=9; 10-20%, n=3; >20%, n=4; all, n=16). * P < .05, ** P < .01. Data represent mean ± SEM with statistical analysis performed using the Wilcoxon signed-rank test. VA, visual acuity; NLP, no light perception; LP, light perception; HM, hand motion; GCC, ganglion cell complex; VFI, visual field index; MD, mean deviation.

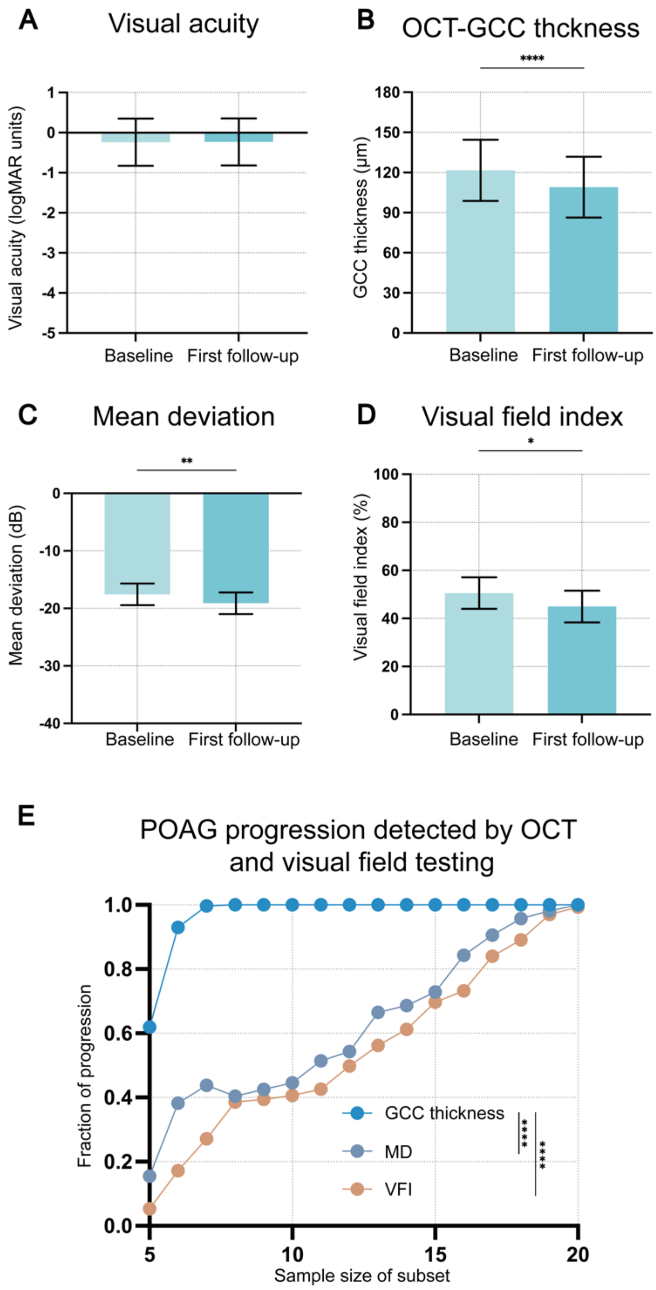

Extended Figure 11. Longitudinal changes in visual and structural parameters in POAG. (**A-D**) Comparison of visual acuity, OCT-measured GCC thickness, MD and VFI between follow-ups in POAG patients (n=25). (**E**) POAG progression detected by OCT and visual field testing between follow-ups. Significant difference between GCC thickness and MD (for each n between 5 and 20, $p < 0.0001$). A-D: data are presented as median (IQR), with statistical analysis performed using the Wilcoxon signed-rank test. E: McNemar's test and Fisher's Exact Test. * $P < 0.05$, ** $P < 0.01$, **** $P < 0.0001$. OCT, optical coherence tomography; GCC, ganglion cell complex; MD, mean deviation; VFI, visual field index; POAG, primary open-angle glaucoma.

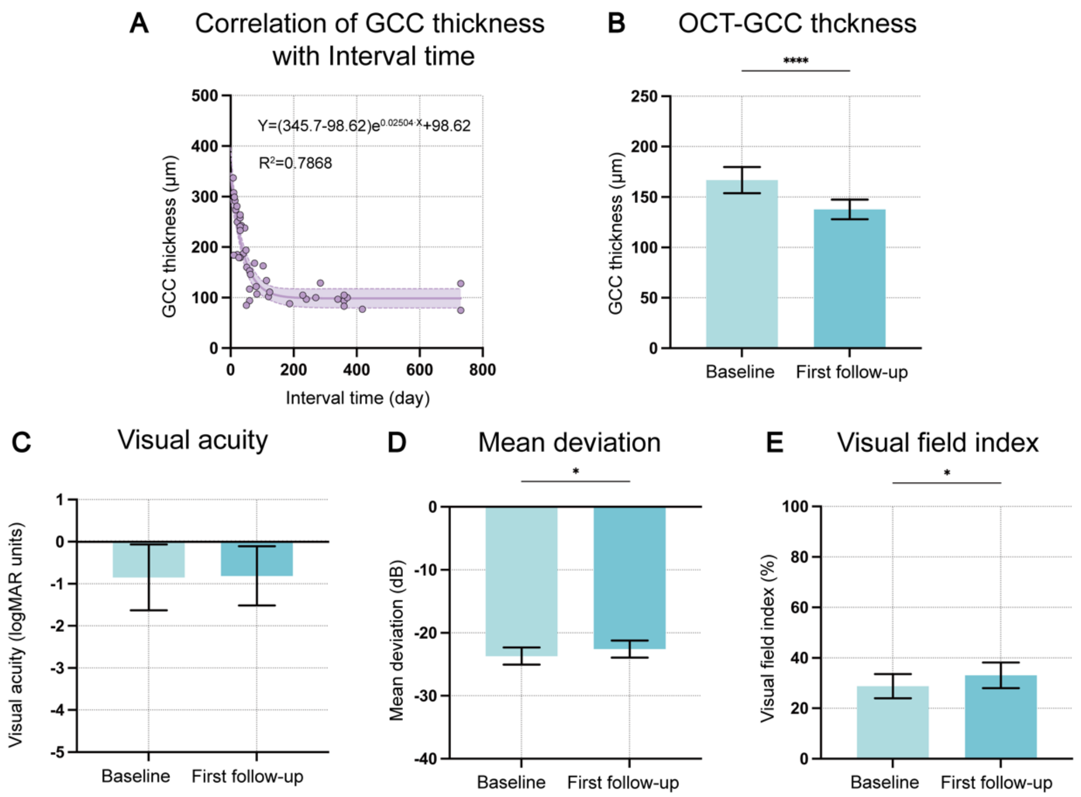

Extended Figure 12. Longitudinal changes in structural and functional visual parameters in AION.

(**A**) Correlation between GCC thickness and interval time in AION patients, modeled with a non-linear regression curve using a "one phase decay" equation. (n=28). (**B**) Comparison of OCT-measured GCC thickness between follow-ups in AION patients (n=28). (**C**) Comparison of visual acuity (logMAR units) between follow-ups in AION patients (n=28). (**D**) Comparison of MD between follow-ups in AION patients (n=28). (**E**) Comparison of VFI between follow-ups in AION patients (n=28). B-E: data are presented as median (IQR), Wilcoxon signed-rank test. * $P < 0.05$, **** $P < 0.0001$. OCT, optical coherence tomography; GCC, ganglion complex cell; MD, mean deviation; VFI, visual field index; AION, anterior ischemic optic neuropathy.

Extended Table 1. Previous clinical trials for TON

Author, Year	Comparative Trial	Biomarker to stratify TON severity（Y/N）			Efficacy of Intervention
		Visual Function	Time Interval	Inner Retinal Thickness	
Yang Gao et al, 2021 (PMID: 33977319)	Y, ETOCD vs conservative treatments.	Y	Y	N	Improved VA, VEP, Retinal artery SO2, the retinal thickness and retinal vessel density were notably better.
Jia Li et al, 2021 (PMID: 33091914)	Y, stem cell transplantation after decompression vs only decompression.	Y	Y	N	No difference.
Hsin-Hung Chen et al, 2020 (PMID: 31800551)	Y, surgery vs steroid.	N	N	N	No difference.
Mohsen Bahmani Kashkouli et al, 2018 (PMID: 28986670)	Y, EPO vs steroid vs observation.	Y	Y	N	No difference.
M Reza Razeghinejad et al, 2010 (PMID: 20649490)	Y, methylprednisolone vs methylprednisolone + Levodopa-Carbidopa.	Y	N	N	No difference in VA or PRL.
Ashok K Gupta et al, 2007 (PMID: 17303257)	N, only compared with baseline.	N	N	N	Patients presenting early and those who were operated early in the course of their illness showed a better recovery.
Morteza Entezari et al, 2007 (PMID: 17265030)	Y, RCT, steroid vs observation.	Y	Y	N	No difference in VA.
Nitza Goldenberg-Cohen et al, 2004 (PMID: 14970795)	Y, steroid vs surgery vs observation.	Y	N	N	No difference in VA.

Yang Yang et al, 2006 (PMID: 17041495)	N, only compared with baseline.	N	N	N	Significant visual acuity improvement occurred in eight (nine eyes) out of our 12 patients after surgery.
Wanicha Chuenkongkaew et al, 2002 (PMID: 12188391)	Y, RCT, methylprednisolone vs dexamethasone.	Y	Y	N	No difference in VA.
Kitthaweesin et al, 2001 (PMID: 11560210)	Y, RCT, methylprednisolone vs dexamethasone.	Y	Y	N	No difference in VA.
L A Levin et al, 1999 (PMID: 10406604)	Y, observation vs steroid vs surgery.	N	Y	N	No difference in VA.
S Mine et al, 1999 (PMID: 10071683)	Y, steroid vs surgery.	Y	N	N	When initial visual acuity was HM or better, vision improved significantly more in patients with surgery than in those without surgery.
A Carta et al, 2006 (PMID: 17191191)	N, only compared with baseline.	Y	Y	N	No difference in VA.
Ching-Hua Hsieh et al, 2004 (PMID: 15187745)	Y, observation vs steroid vs surgery.	Y	N	N	No difference in VA.
I-Li Lai et al, 2016 (PMID: 26808764)	N, only compared with baseline.	Y	N	N	Comparing the influence of other factors on the final VA, it was found that except for VA and extraocular movement, there was no clear effect.
Kok Foo Lee et al, 2010 (PMID: 22553547)	Y, observation vs steroid.	Y	N	N	Patients treated with intravenous followed by oral corticosteroids have better VA compared to patients under conservative management.

Nazife Sefi-Yurdakul et al, 2018 (PMID: 28681279)	Y, observation vs steroid.	N	N	N	Final VA was improved in both the hormone-treated and observation groups, but final VA was better in the observation group than in the hormone-treated group.
Sanjeeta Sitaula et al, 2017 (PMID: 29467804)	Y, observation vs low-dose steroid vs high-dose steroid.	Y	Y	N	Steroid has an effect on the recovery of visual acuity regardless of the high or low dose
Wentao Yan et al, 2017 (PMID: 27267448)	Y, VA of different types of optic canal fractures before and after surgery.	N	Y	N	Surgical efficacy existed among different groups with different kind of OCF.
Chien-Tzung Chen et al, 2007 (PMID: 17251830)	N, only compared with baseline.	Y	Y	N	This article concluded that the outcome of decompression surgery was only related to the age and was not related to the baseline VA, OCF, and the interval time.
Min Chen et al, 2018 (PMID: 30250525)	Y, steroid vs steroid + surgery.	N	N	N	No comparison was made, only the number of people with different vision was shown, and the overall improvement was achieved.
E Emanuelli et al, 2015 (PMID: 25472815)	Y, surgery vs steroid + surgery.	Y	Y	N	Whether it was surgery or steroid therapy, the patients who received treatment within 12 hours after injury had better VA than those who received treatment beyond 12 hours, and the final VA was inversely proportional to the interval time.
Nitza Goldenberg-Cohen et al, 2004 (PMID: 14970795)	Y, observation vs high-dose steroid vs surgery.	Y	N	N	The sample size was too small, there were too many groups, all were final visual presentations, and no statistical results were obtained.

Bo Yu et al, 2016 (PMID: 27965891)	Y, time interval within 3 days vs 3-7 days vs without 7 days	Y	Y	N	In terms of final VA improvement, more patients who underwent surgery within 3 days than after 7 days had improved VA, but there was no statistical difference in the rest of the time.
Zhen-Hua He et al, 2016 (PMID: 28088936)	N, only compared with baseline.	N	N	N	No difference in VA.
Zhenhua He et al, 2015 (PMID: 26005018)	N, only compared with baseline.	N	N	N	There was no control group, but 53.8% of the patients had improved VA after surgery. The better the improvement of VA the shorter the latency and the larger the amplitude of VEP.
I-Li Lai et al, 2018 (PMID: 29705230)	Y, receiving treatment vs not receiving treatment.	Y	N	N	VA improved in 34.2% of treated patients and none of untreated patients, and patients with an initial IOP (17–23 mmHg) were more likely to recover.
H Li et al, 2008 (PMID: 18439333)	Y, steroid vs steroid + surgery.	N	N	N	There was no statistically significant difference in the number of VA improvement between the steroid group and the surgical + steroid group, but the number of patients with immediate blindness improved less than those with gradual visual loss.
H B Li et al, 2007 (PMID: 18076430)	N, only compared with baseline.	Y	N	N	No difference in VA.
Ying-Jie Ma et al, 2018 (PMID: 30046543)	N, only compared with baseline.	N	N	N	Multiple linear regression analyses of postoperative visual acuity and the degree of visual improvement were performed, yielding

					statistically significant results for age, optic canal fracture, and interval time.
Michael Sosin et al, 2016 (PMID: 26710028)	N, only compared with baseline.	N	N	N	Only the number of people with different visual outcomes in different subgroups (age, sex, optic nerve fracture, length of hospital stay, etc.) is reported.
Qin-Tai Yang et al, 2012 (PMID: 22673265)	N, only compared with baseline (surgery).	N	N	N	The proportion of patients with postoperative VA improvement was 40.6%. The initial VA and interval time (3days) had a statistically significant effect on VA improvement, but the interval time (7days) had no effect.
Wen-Guei Yang et al, 2004 (PMID: 14676697)	Y, steroid vs steroid + surgery.	N	N	N	No difference in VA.
Bo Yu et al, 2018 (PMID: 29940917)	N, only compared with baseline (surgery).	N	N	N	Postoperative VA was improved in 54.84% of the total number of patients.
Mohsen Bahmani Kashkouli et al, 2011 (PMID: 20890611)	Y, EPO vs observation.	N	N	N	There was no significant difference in the initial VA between the observation group and the EPO treatment group, but the final VA of the observation group was better than that of the EPO treatment group after treatment.
Morteza Entezari et al, 2014 (PMID: 24986593)	N, only compared with baseline (EPO).	N	N	N	The BCVA (LogMAR) was statistically significant compared with the initial visual acuity at both 1-month and 3-month follow-up.
Mohammad Ahmad Rashad et al, 2018	N, only compared with baseline (EPO).	N	N	N	The patients' VA after the first vitreous injection of EPO was statistically

(PMID: 30647957)					significant compared to the initial VA, but the second injection was not statistically significant compared to the first.
V Lee et al, 2010 (PMID: 19407847)	Y, no treatment vs treatment (steroids or surgery).	N	N	N	No difference in VA.

Extended Table 2. Comparative analysis of functional and structural parameters between the injured eye and the contralateral eye in patients with unilateral TON

Functional and structural parameters	Injured eye, Median [IQR]	Contralateral eye[A], Median [IQR]
VAcc	-2.70 [-4.70~-1.10]	0.00 [0.00~0.00]
RNFL thickness (μm)	96.00 [74.00~107.00]	106.00 [100.00~112.00]
GCC relative thickness (%)	85.92 [69.82~93.98]	100.00 [100.00~100.00]
VFI (%)	0.00 [0.00~28.00]	97.00 [94.00~99.00]
MD (dB)	-30.17 [-33.13~-21.18]	-2.79 [-5.01~-1.50]
P-VEP P100 amplitude (μv)	2.40 [1.20~4.20]	7.60 [4.50~11.70]
P-VEP P100 latency (ms)	122.00 [102.00~145.00]	104.00 [100.00~106.00]

[A] The best corrected VA of the contralateral eye ≥ 20/20 and the patients have no other ocular diseases or systemic diseases that may affect the eyes.

Extended Table 3. Demographic and Clinical Characteristics of Patients with Traumatic Optic Neuropathy Receiving Pharmaceutical Treatments

Characteristics	No. (%)
**Gender**	
Male	18(85.7)
Female	3(14.3)
**Median age (IQR), years**	21(35)
<18	9(42.9)
18-44	6(28.6)
45-60	4(19.1)
>60	2(9.5)
**The time interval, Median Time (IQR), days** [A]	30(53)
<7	5(23.8)
7-14	3(14.3)
15-30	5(23.8)
>30	8(38.1)
**Injury eye**	
Right	12(57.1)
Left	8(38.1)
Both	1(4.8)
**Injury cause**	
Traffic accident	3(14.3)
Fall	16(76.2)
Others	2(9.5)

[A] The interval between injury and ophthalmic examination.
